# New particle formation from isoprene under upper-tropospheric conditions

**DOI:** 10.1038/s41586-024-08196-0

**Published:** 2024-12-04

**Authors:** Jiali Shen, Douglas M. Russell, Jenna DeVivo, Felix Kunkler, Rima Baalbaki, Bernhard Mentler, Wiebke Scholz, Wenjuan Yu, Lucía Caudillo-Plath, Eva Sommer, Emelda Ahongshangbam, Dina Alfaouri, João Almeida, Antonio Amorim, Lisa J. Beck, Hannah Beckmann, Moritz Berntheusel, Nirvan Bhattacharyya, Manjula R. Canagaratna, Anouck Chassaing, Romulo Cruz-Simbron, Lubna Dada, Jonathan Duplissy, Hamish Gordon, Manuel Granzin, Lena Große Schute, Martin Heinritzi, Siddharth Iyer, Hannah Klebach, Timm Krüger, Andreas Kürten, Markus Lampimäki, Lu Liu, Brandon Lopez, Monica Martinez, Aleksandra Morawiec, Antti Onnela, Maija Peltola, Pedro Rato, Mago Reza, Sarah Richter, Birte Rörup, Milin Kaniyodical Sebastian, Mario Simon, Mihnea Surdu, Kalju Tamme, Roseline C. Thakur, António Tomé, Yandong Tong, Jens Top, Nsikanabasi Silas Umo, Gabriela Unfer, Lejish Vettikkat, Jakob Weissbacher, Christos Xenofontos, Boxing Yang, Marcel Zauner-Wieczorek, Jiangyi Zhang, Zhensen Zheng, Urs Baltensperger, Theodoros Christoudias, Richard C. Flagan, Imad El Haddad, Heikki Junninen, Ottmar Möhler, Ilona Riipinen, Urs Rohner, Siegfried Schobesberger, Rainer Volkamer, Paul M. Winkler, Armin Hansel, Katrianne Lehtipalo, Neil M. Donahue, Jos Lelieveld, Hartwig Harder, Markku Kulmala, Doug R. Worsnop, Jasper Kirkby, Joachim Curtius, Xu-Cheng He

**Affiliations:** 1 Institute for Atmospheric and Earth System Research/Physics, Faculty of Science, University of Helsinki, Helsinki, Finland; 2 Helsinki Institute of Physics, University of Helsinki, Helsinki, Finland; 3 Institute for Atmospheric and Environmental Sciences, Goethe University Frankfurt, Frankfurt am Main, Germany; 4 Center for Atmospheric Particle Studies, Carnegie Mellon University, Pittsburgh, PA USA; 5 Atmospheric Chemistry Department, Max Planck Institute for Chemistry, Mainz, Germany; 6 Institute for Ion Physics and Applied Physics, University of Innsbruck, Innsbruck, Austria; 7 CERN, the European Organization for Nuclear Research, Geneva, Switzerland; 8 Faculty of Physics, University of Vienna, Wien, Austria; 9 Department of Chemistry, University of Helsinki, Helsinki, Finland; 10 CENTRA and Faculdade de Ciências da Universidade de Lisboa, Lisboa, Portugal; 11 Department of Environmental Physics, University of Tartu, Tartu, Estonia; 12 Aerodyne Research Inc., Billerica, MA USA; 13 Department of Environmental Science, Stockholm University, Stockholm, Sweden; 14 Department of Chemistry, University of Colorado Boulder, Boulder, CO USA; 15 Cooperative Institute for Research in Environmental Sciences, University of Colorado Boulder, Boulder, CO USA; 16 Laboratory of Atmospheric Chemistry, Paul Scherrer Institute, Villigen, Switzerland; 17 Aerosol Physics Laboratory, Faculty of Engineering and Natural Sciences, Tampere University, Tampere, Finland; 18 Department of Chemical Engineering, Carnegie Mellon University, Pittsburgh, PA USA; 19 Institute of Meteorology and Climate Research, Atmospheric Aerosol Research, Karlsruhe Institute of Technology, Karlsruhe, Germany; 20 Instituto Dom Luiz (IDL), Universidade da Beira Interior, Covilhã, Portugal; 21 Atmospheric Microphysics Department, Leibniz Institute for Tropospheric Research (TROPOS), Leipzig, Germany; 22 Department of Technical Physics, University of Eastern Finland, Kuopio, Finland; 23 Climate and Atmosphere Research Centre (CARE-C), The Cyprus Institute, Nicosia, Cyprus; 24 IONICON Analytik GmbH, Innsbruck, Austria; 25 Division of Chemistry and Chemical Engineering, California Institute of Technology, Pasadena, CA USA; 26 TOFWERK, Thun, Switzerland; 27 Finnish Meteorological Institute, Helsinki, Finland; 28 Department of Engineering and Public Policy, Carnegie Mellon University, Pittsburgh, PA USA; 29 Department of Chemistry, Carnegie Mellon University, Pittsburgh, PA USA; 30 Joint International Research Laboratory of Atmospheric and Earth System Sciences, School of Atmospheric Sciences, Nanjing University, Nanjing, China; 31 Aerosol and Haze Laboratory, Beijing Advanced Innovation Center for Soft Matter Science and Engineering, Beijing University of Chemical Technology, Beijing, China; 32 Yusuf Hamied Department of Chemistry, University of Cambridge, Cambridge, UK

**Keywords:** Atmospheric science, Climate change

## Abstract

Aircraft observations have revealed ubiquitous new particle formation in the tropical upper troposphere over the Amazon^1,2^ and the Atlantic and Pacific oceans^3,4^. Although the vapours involved remain unknown, recent satellite observations have revealed surprisingly high night-time isoprene mixing ratios of up to 1 part per billion by volume (ppbv) in the tropical upper troposphere^5^. Here, in experiments performed with the CERN CLOUD (Cosmics Leaving Outdoor Droplets) chamber, we report new particle formation initiated by the reaction of hydroxyl radicals with isoprene at upper-tropospheric temperatures of −30 °C and −50 °C. We find that isoprene-oxygenated organic molecules (IP-OOM) nucleate at concentrations found in the upper troposphere, without requiring any more vapours. Moreover, the nucleation rates are enhanced 100-fold by extremely low concentrations of sulfuric acid or iodine oxoacids above 10^5^ cm^−3^, reaching rates around 30 cm^−3^ s^−1^ at acid concentrations of 10^6^ cm^−3^. Our measurements show that nucleation involves sequential addition of IP-OOM, together with zero or one acid molecule in the embryonic molecular clusters. IP-OOM also drive rapid particle growth at 3–60 nm h^−1^. We find that rapid nucleation and growth rates persist in the presence of NO_*x*_ at upper-tropospheric concentrations from lightning. Our laboratory measurements show that isoprene emitted by rainforests may drive rapid new particle formation in extensive regions of the tropical upper troposphere^1,2^, resulting in tens of thousands of particles per cubic centimetre.

## Abstract (web summary)

Experiments performed in the CERN CLOUD chamber show that, under upper-tropospheric conditions, new atmospheric particle formation may be initiated by the reaction of hydroxyl radicals with isoprene emitted by rainforests.

## Main

Aerosol particles are important for climate because they scatter and absorb incoming solar radiation and seed cloud droplets by acting as cloud condensation nuclei (CCN). More CCN make clouds more reflective and may increase their extent and lifetime. Around half of CCN globally, and almost all in the upper troposphere^6^, arise from new particle formation, which involves the spontaneous condensation of low-volatility vapours in the atmosphere to form liquid or solid particles (particle nucleation). The initial stable molecular clusters form at diameters slightly above 1 nm. To become CCN, the new particles should not be scavenged by pre-existing aerosol but grow by further vapour condensation to a size of around 50 nm and larger (particle growth). Although new particle formation has been extensively studied at ground-based sites^7^, little is known about the precursor vapours responsible for new particles in the remote upper troposphere and in marine regions. In particular, high concentrations of freshly formed particles are observed in the upper free troposphere over the Amazon^1,2^ and the tropical Atlantic and Pacific oceans^3,4^. Chemical-transport models indicate that new particle formation persists across the tropical upper troposphere over a latitude band covering about 40% of Earth’s surface^3^ and provides a global supply of CCN for low-altitude clouds in the sub-tropics and tropics^3,8^. However, the source of these particles has remained a puzzle for the past 20 years.

Early studies proposed that convective clouds could transport vapours from the boundary layer and form new particles in cold cloud outflows at high altitudes^4,9^. In the absence of an established mechanism, it was suggested that the oxidation products of isoprene (C_5_H_8_) could contribute to the high mass concentrations of freshly formed particles observed in the upper troposphere over the Amazon^10^. Recent modelling studies have speculated that pure biogenic new particle formation from monoterpenes is the source of these particles^11,12^, but insufficient monoterpene concentrations have been found to account for the high particle-number concentrations^13^.

On the other hand, recent satellite observations have revealed high concentrations of isoprene in the upper troposphere over tropical South America, Central Africa and Southeast Asia, reaching up to around 1 ppbv during night-time^5^ (1 ppbv is equivalent to around 0.6 × 10^10^ molecules cm^−3^ at 10 km altitude and 2.5 × 10^10^ molecules cm^−3^ at 0 km). The isoprene concentrations fall during daytime owing to high hydroxyl radical (OH) concentrations, which result in an isoprene lifetime of around 1–2 h. Furthermore, observations at 5,240 m altitude in the Bolivian Andes have found isoprene oxidation products in both gas and particle phases in air masses originating from the Amazon free troposphere^14^. Isoprene is emitted by vegetation, especially deciduous and broad-leaved evergreen trees, and is the most abundant hydrocarbon emitted into the atmosphere, after methane^15^. Median isoprene mixing ratios in the Amazon rainforest vary between 0.5 and 2.0 ppbv at night and 2.0–6.0 ppbv during the day^16^. Modelling studies show that isoprene is efficiently transported from the tropical boundary layer to the upper troposphere in deep convective clouds^17^.

Isoprene influences the oxidation capacity of the atmosphere^18,19^ and contributes to the formation of secondary organic aerosol^20–24^ particle mass, which—in turn—affects the climate^25,26^. The contribution of isoprene to secondary organic aerosol particle mass is at present believed to be dominated by the reactive uptake of isoprene dihydroxy epoxide (IEPOX)^21,27^ and other IP-OOM^28^. Lower contributions are thought to arise from the condensation of low-volatility compounds formed during oxidation of the first-generation product, isoprene hydroxy hydroperoxide (ISOPOOH)^29–31^. IEPOX and ISOPOOH are isomers of C_5_H_10_O_3_ and cannot be separated by normal chemical-ionization mass spectrometry. The ability of isoprene to form new particles is considered negligible^24^ and, moreover, isoprene inhibits new particle formation from monoterpenes under boundary-layer conditions^32–34^. However, it has so far remained unknown whether IP-OOM^35^ can form new particles in the upper troposphere, where it is extremely cold and scavenging losses are small, and—if so—what are the associated nucleation and growth rates.

## CLOUD experiment

Here we report experiments performed in the CERN CLOUD chamber^36^ to study new particle formation from the reaction of OH with isoprene at upper-tropospheric concentrations and temperatures of −30 °C and −50 °C. The experiments were performed during the CLOUD15 and CLOUD16 campaigns, September–November 2022 and 2023, respectively. Before injection into the chamber, the isoprene vapour was passed through a cryo-trap at −53 °C to eliminate low-volatility contaminants (as confirmed by mass-spectrometer measurements). Further details of the CLOUD facility and its analysing instruments are provided in Methods.

The range of experimental parameters is summarized in Extended Data Table 1, together with the ambient upper-tropospheric conditions over the Amazon measured by Curtius et al.^13^ during research flight (RF) 19. To maximize the IP-OOM detection efficiency, we combined the measurements of three mass spectrometers that use ammonium, nitrate and bromide chemical-ionization (see Methods for further details), whereas the research flight uses nitrate ionization alone. In general, there is good overlap of the CLOUD experiments with the ambient conditions for this single flight (see Methods for further discussion). We note that, although the CLOUD chamber operates at atmospheric pressure, this does not affect the simulation of particle-nucleation dynamics at upper-tropospheric conditions. We performed experiments with nitrogen oxide (NO) concentrations varied between zero and around 7 × 10^9^ cm^−3^, characteristic of the outflow from electrified deep convective clouds. Trace amounts of sulfuric acid (H_2_SO_4_), methanesulfonic acid (CH_3_SO_3_H) and iodine oxoacids^37^ (HIO_*x*_, *x* = 2, 3) also exist in the upper troposphere from the oxidation of vapours such as sulfur dioxide (SO_2_), dimethyl sulfide (DMS; CH_3_SCH_3_) and iodine (I_2_), respectively. For some experiments, therefore, we introduced upper-tropospheric concentrations of sulfuric acid or iodine oxoacids to explore their interactions with IP-OOM.

## IP-OOM

Hydroxyl radicals preferentially attack one of the terminal carbon atoms of isoprene, forming isoprene peroxy radicals (ISOPOO, C_5_H_8_(OH)(OO)). In the absence of NO, ISOPOO reacts with hydroperoxy radicals (HO_2_) to form ISOPOOH (C_5_H_8_(OH)(OOH))^35,38^. In the presence of NO, ISOPOO also reacts to form isoprene hydroxy nitrate (IHN, C_5_H_8_(OH)(ONO_2_)) and further products^35^. Further reaction of IHN with OH forms isoprene dihydroxy dinitrate (C_5_H_8_(OH)_2_(ONO_2_)_2_) and other second-generation products containing one or two nitrogen atoms. Further reactions of ISOPOOH with OH include the formation of its isomer, IEPOX^21^, as well as second-generation products containing zero or one nitrogen atom.

Oxidation by hydroxyl radicals of ISOPOOH^29,30^, IEPOX^21,39,40^ and IHN are the main channels that feed the second-generation IP-OOM, IP_0-2N_. Because the volatility of IP-OOM largely depends on their oxygen number^41^, we define IP_0-2N_ as C_*i*_H_*j*_O_*k*_N_*l*_ with the requirements that *i*, *j* ≥ 4, *l* = 0–2 and with a minimum oxygen number that takes the nitrogen content into account. As a nitrate group (-O-NO_2_) is considered to decrease the volatility of an organic compound by about the same factor as a hydroxyl group (-OH)^41^, we require (*k* − 2*l*) ≥ 4. We define IP_0N_, IP_1N_ and IP_2N_ as IP-OOM containing 0, 1 or 2 nitrogen atoms, respectively. In our study, therefore, IP_0-2N_ (= IP_0N_ + IP_1N_ + IP_2N_) excludes ISOPOOH and IEPOX but may include some first-generation IP-OOM from ISOPOO^35^. The IP_0-2N_ include IP-OOM monomers with up to five carbon atoms (C_5_) and dimers with up to ten carbons (C_10_), formed by reactions between two isoprene peroxy radicals (RO_2_), which produce covalently bound molecules.

**Extended Data Fig. 1:**
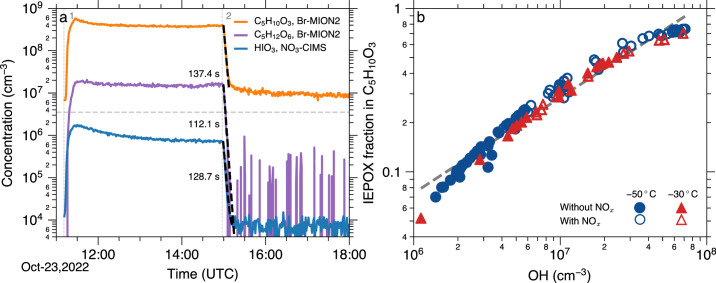
Wall-loss rates of C_5_H_10_O_3_, C_5_H_12_O_6_ and HIO_3_ at −30 °C and the IEPOX fraction of C_5_H_10_O_3_ at −30 °C and −50 °C. **a**, Wall-loss lifetimes (1/(loss rate)) of C_5_H_10_O_3_, C_5_H_12_O_6_ and HIO_3_ at 100% mixing fan speed (increased loss rate). At stage 1, the mixing fan speed is set to 12% (standard operation). At stage 2, the mixing fan speed is set to 100% to increase the wall-loss rate. All three vapours have wall-loss rates consistent with that measured for H_2_SO_4_ (not shown), indicating irreversible loss on wall impact. **b**, Simulation of the fraction of IEPOX in C_5_H_10_O_3_ using the reduced Wennberg et al. mechanism^35^. The dashed grey line is a fit to the data of the form ${\rm{IEPOX}}\,{\rm{fraction}}={10}^{(0.58\times {\log }_{10}([{\rm{OH}}])-4.6)}$. Our simulation predicts that roughly 30% of the measured C_5_H_10_O_3_ signal is IEPOX under the conditions of panel **a** and that both the ISOPOOH and IEPOX (summed as C_5_H_10_O_3_) are lost to the chamber wall on impact, at −30 °C and at −50 °C. The experimental conditions for panel **a** are: isoprene = 0.13 ppbv (3.7 × 10^9^ cm^−3^), O_3_ = 30 ppbv (8.6 × 10^11^ cm^−3^), I_2_ = 3.1 × 10^7^ cm^−3^, SO_2_ = 3 × 10^8^ cm^−3^, OH = 8.9 × 10^6^ cm^−3^, HO_2_ = 1.8 × 10^8^ cm^−3^, RH = 57% and temperature = −30 °C. The experimental conditions for panel **b** are: isoprene = 0.04–1.50 ppbv (0.1–4.2 × 10^10^ cm^−3^), O_3_ = 1–590 ppbv (3.7 × 10^10^ to 1.8 × 10^13^ cm^−3^), I_2_ = 0–7.5 × 10^7^ cm^−3^, SO_2_ = 0–4.6 × 10^9^ cm^−3^, OH = 0.11–6.90 × 10^7^ cm^−3^, HO_2_ = 0.6–17.0 × 10^8^ cm^−3^, HO_2_/OH ratio = 11–118, NO = 0–0.22 ppbv, NO_2_ = 0–0.77 ppbv, RH = 29–70% and temperature = −30 °C and −50 °C.

**Extended Data Fig. 2:**
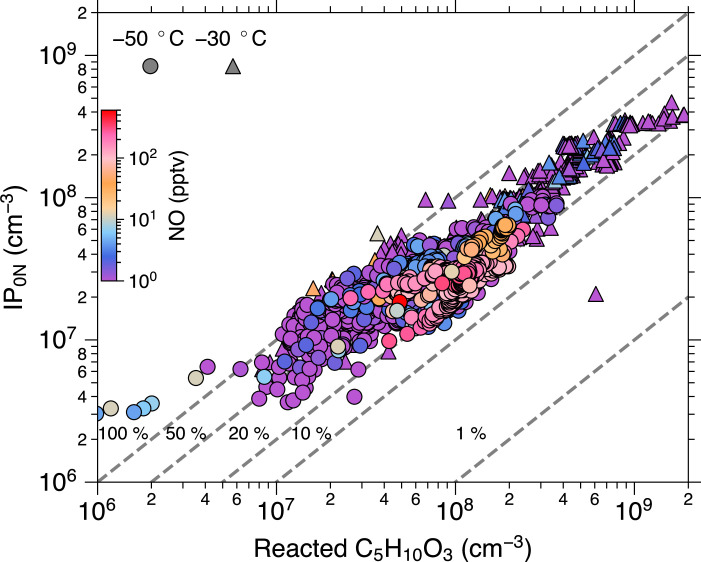
IP_0N_ yield from OH reaction with C_5_H_10_O_3_. Measured IP_0N_ concentration versus reacted C_5_H_10_O_3_ (see Methods for details). The dashed lines show the predictions for several IP_0N_ molar yields; a yield of 100% implies that each OH reaction with C_5_H_10_O_3_ produces one IP_0N_ molecule. The data indicate that the yield is between 20% and 50%, with an overall systematic uncertainty of a factor of two. When NO_*x*_ is added to the system, the yield of IP_0N_ is reduced, owing to NO terminating isoprene peroxy radicals. The ratios of IEPOX and ISOPOOH in C_5_H_10_O_3_ are determined from the measured OH concentrations and the fit to the simulation shown in Extended Data Fig. 1b. For simplification, we apply a general reaction-rate coefficient of 10^−10^ and 10^−11^ cm^3^ s^−1^ for the ISOPOOH + OH and IEPOX + OH reactions, respectively^35^. The experimental conditions are: isoprene = 0.04–1.50 ppbv (0.1–4.2 × 10^10^ cm^−3^), O_3_ = 1–590 ppbv (3.7 × 10^10^ to 1.8 × 10^13^ cm^−3^), I_2_ = 0–7.5 × 10^7^ cm^−3^, SO_2_ = 0–4.6 × 10^9^ cm^−3^, OH = 0.11–6.90 × 10^7^ cm^−3^, HO_2_ = 0.6–18.0 × 10^8^ cm^−3^, HO_2_/OH ratio = 11–117, NO = 0–0.22 ppbv, NO_2_ = 0–0.77 ppbv, RH = 29–70% and temperature = −30 °C and −50 °C.

As with other condensable vapours, both ISOPOOH and IEPOX deposit irreversibly on the CLOUD chamber walls at −50 °C (ref. ^42^) and also at −30 °C. The latter is confirmed by our measurements of a wall-loss lifetime for C_5_H_10_O_3_ at −30 °C, which indicates irreversible loss on impact (Extended Data Fig. 1a). Although we cannot distinguish ISOPOOH from IEPOX, chemical model calculations^35,43^ indicate that more than 80% of our C_5_H_10_O_3_ signal is ISOPOOH at 5.5 × 10^6^ cm^−3^ OH (Methods and Extended Data Fig. 1b). Assuming this ISOPOOH fraction, we measure the total molar yield of IP_0N_ from the reaction of hydroxyl radicals with C_5_H_10_O_3_ in the absence of nitrogen oxides (NO_*x*_) to be 46% at −30 °C and 55% at −50 °C, with a systematic uncertainty of a factor of two (Extended Data Fig. 2). In the presence of NO_*x*_, the IP_0N_ yield falls to around 38% owing to RO_2_ termination by NO or by further reactions that produce IP_1N_ and IP_2N_ (Extended Data Fig. 2). At upper-tropospheric NO concentrations of up to 7 × 10^9^ cm^−3^, we measure the nitrate IP-OOM fraction to be IP_1-2N_/IP_0-2N_ = 23–88%, in which IP_1-2N_ = IP_1N_ + IP_2N_.

## Example experiments

**Fig. 1:**
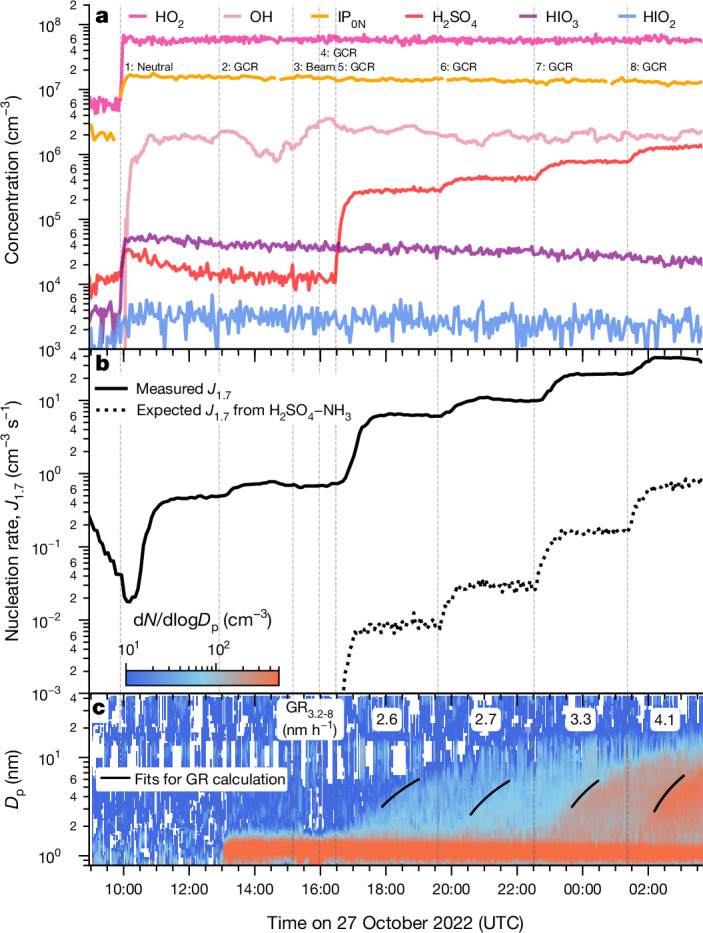
Example new particle formation experiment from IP-OOM at −50 °C, without NO_*x*_. **a**–**c**, Evolution of vapour concentrations (**a**), particle-nucleation rates at 1.7 nm, *J*_1.7_ (**b**), and naturally charged negative particle number size distribution (d*N*/dlog*D*_p_) and growth rates measured between 3.2 and 8.0 nm, GR_3.2-8_ (nm h^−1^), for total (naturally charged + neutral) particles (**c**). The black lines in **c** depict the linear fits of 50% appearance time of particles between 3.2 and 8.0 nm. The vertical dashed lines and labels indicate the start of a new stage, at which the experimental conditions were adjusted. Trace sulfuric and iodic acid contaminants are present at the start of the run at concentrations of 1–5 × 10^4^ cm^−^^3^. Sulfur dioxide is injected and progressively increased during stages 5–8, which produces steps in H_2_SO_4_. The dotted black curve in **b** shows the expected H_2_SO_4_–NH_3_ nucleation rate, conservatively assuming that NH_3_ is present at the 4 pptv limit of detection. Stages 1 and 3 are, respectively, under neutral (ion-free) and beam (ion-enhanced) conditions, whereas all of the other stages are under galactic cosmic ray (GCR; natural ion concentrations) conditions. The experimental conditions are: isoprene = 0.20–0.41 ppbv (6.2–13.0 × 10^9^ cm^−3^), O_3_ = 84–96 ppbv (2.6–3.0 × 10^12^ cm^−3^), I_2_ = 3.7–23.0 × 10^5^ cm^−3^, SO_2_ = 0–4 × 10^9^ cm^−3^, OH = 1–3 × 10^6^ cm^−3^, HO_2_ = 4.7–6.0 × 10^7^ cm^−3^, HO_2_/OH ratio = 18–37, RH = 62%, NO less than limit of detection (7 pptv) and temperature = −49 °C.

A typical run sequence in the absence of NO_*x*_ is shown in Fig. 1. The experiment started at stage 1 by setting the internal mixing fans from 100% (high wall-loss rate) to 12% (standard operation) and switching on the ultraviolet (UV) light, which photolysed ozone to produce 2 × 10^6^ cm^−3^ OH and 6 × 10^7^ cm^−3^ HO_2_ radicals. Stages 1, 2 and 3 were, respectively, under different ionization conditions: neutral (all ions swept from the chamber by high-voltage electrodes); natural ionization from galactic cosmic rays (high voltage switched off); and pion beam (upper-tropospheric ion concentrations). The nucleation rate, *J*_1.7_, is the measured flux of particles passing a 1.7-nm threshold size. Because *J*_1.7_ varied little between these stages (Fig. 1b), it indicates relatively little sensitivity to ion concentrations. Before stage 5, the nucleation rate was 0.48–0.73 cm^−3^ s^−1^, whereas IP_0N_ were around 1.6 × 10^7^ cm^−3^ and sulfuric acid and iodine oxoacids were both below 10^5^ cm^−3^. These nucleation rates far exceed those expected for HIO_*x*_ and H_2_SO_4_, conservatively assuming 1 × 10^8^ cm^−3^ contaminant ammonia (NH_3_) (refs. ^37,44,45^). As no other condensable vapours were present, this experiment shows that IP-OOM form new particles under upper-tropospheric conditions at −50 °C.

During stages 5–8, SO_2_ was introduced into the chamber in steps, while keeping all other experimental conditions fixed (Fig. 1a). This increased the H_2_SO_4_ concentration from 1.5 × 10^4^ cm^−3^ to between 2.8 × 10^5^ and 1.3 × 10^6^ cm^−3^. Despite these extremely low acid concentrations, which are equivalent to between 0.05 and 0.2 parts per trillion by volume (pptv) at 10 km altitude, the nucleation rate increased from 6.2 to 37 cm^−3^ s^−1^ between stages 5 and 8, respectively (Fig. 1b). Once again, these nucleation rates far exceed those expected for H_2_SO_4_–NH_3_. The particle growth rates between 3.2 and 8.0 nm increased from 2.6 to 4.1 nm h^−1^ between stages 5 and 8 (Fig. 1c). Because the expected particle growth rate for 1.3 × 10^6^ cm^−3^ acid is around 0.15 nm h^−1^ (ref. ^46^), these high growth rates must largely result from condensation of IP_0N_.

**Extended Data Fig. 3:**
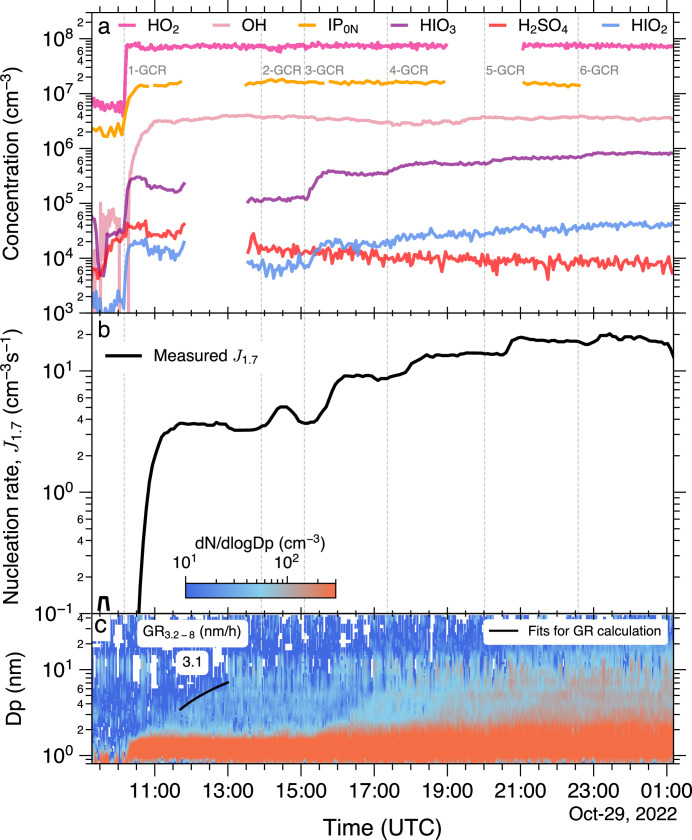
Example new particle formation experiment at −50 °C with added HIO_*x*_. Evolution of vapour concentrations (**a**), particle-nucleation rates at 1.7 nm, *J*_1.7_ (**b**), and naturally charged particle number size distribution (d*N*/dlog*D*_p_) and growth rate measured between 3.2 and 8.0 nm, GR_3.2-8_ (nm h^−1^) (**c**). The black line in panel **c** depicts the linear fit of 50% appearance time of particles between 3.2 and 8.0 nm. The expected nucleation rates for HIO_*x*_ at the kinetic limit are less than 2 × 10^−4^ cm^−3^ s^−1^. The results show that HIO_*x*_ enhances IP_0N_ nucleation in a similar way as H_2_SO_4_. Discontinuous lines show missing data. The experimental conditions are: isoprene = 0.07–0.16 ppbv (2.1–4.9 × 10^9^ cm^−3^), O_3_ = 95 ppbv (3 × 10^12^ cm^−3^), I_2_ = 0.4–6.7 × 10^7^ cm^−3^, OH = 3.2 × 10^6^ cm^−3^, HO_2_ = 7.4 × 10^7^ cm^−3^, RH = 63% and temperature = −49 °C.

In other experiments, we introduced small concentrations of HIO_*x*_ in the presence of negligible H_2_SO_4_. Extended Data Fig. 3 shows an example at −50 °C. Here IP_0N_ were held constant at 1.6 × 10^7^ cm^−3^ s^−1^ and contaminant H_2_SO_4_ was less than 5.0 × 10^4^ cm^−3^. The HIO_*x*_ concentration was increased in steps from 1.2 × 10^5^ to 8.6 × 10^5^ cm^−3^ and the nucleation rate increased from 3.3 to 18 cm^−3^ s^−1^. These nucleation rates closely match those measured for the IP_0N_–H_2_SO_4_ system, suggesting that either inorganic acid plays a similar role in nucleation by stabilizing the embryonic molecular clusters.

**Extended Data Fig. 4:**
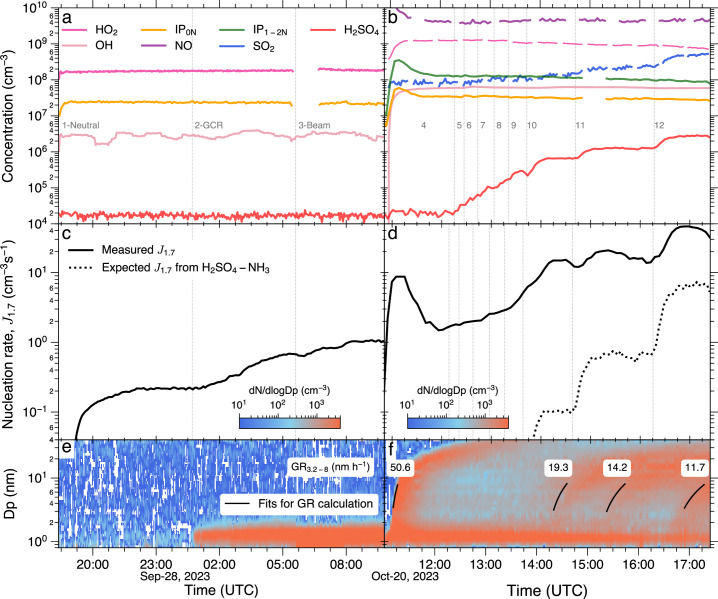
Aerosol formation from pure IP_0N_ products and mixture of IP_0N_, IP_1-2N_ and H_2_SO_4_. Evolution of vapour concentrations (**a**,**b**), particle-nucleation rates at 1.7 nm, *J*_1.7_ (**c**,**d**), and naturally charged particle number size distribution (d*N*/dlog*D*_p_) and growth rates measured between 3.2 and 8.0 nm, GR_3.2-8_ (nm h^−1^) (**e**,**f**). The black lines in panel **f** depict the linear fits of 50% appearance time of particles between 3.2 and 8.0 nm. The results show that pure IP_0N_ aerosol formation is feasible and atmospheric ions can enhance the nucleation rate by a few times. The addition of low concentrations of H_2_SO_4_ further enhances IP_0-2N_ nucleation by up to two orders of magnitude. The vertical dashed lines and labels indicate the start of a new stage in which the experimental conditions were adjusted. The dotted black curve in panel **d** shows the expected H_2_SO_4_–NH_3_ nucleation rate, conservatively assuming that NH_3_ is present at the 4 pptv limit of detection^44,93^. Stages 1 and 3 are, respectively, under neutral (ion-free) and beam (ion-enhanced) conditions, whereas all of the other stages are under galactic cosmic ray (GCR; natural ion concentrations) conditions. The SO_2_ concentration is increased in steps during stages 5–12. The experimental conditions for the right panels are: isoprene = 0.08–0.63 ppbv (2.4–20.0 × 10^9^ cm^−3^), O_3_ = 189 ppbv (6 × 10^12^ cm^−3^), OH = 2.4 × 10^6^ cm^−3^, HO_2_ = 1.8 × 10^8^ cm^−3^, RH = 32% and temperature = −49 °C. The experimental conditions for the left panels are: isoprene = 0.05–0.50 ppbv (1.6–16.0 × 10^9^ cm^−3^), O_3_ = 1.2 ppbv (3.8 × 10^10^ cm^−3^), SO_2_ = 0–1.9 × 10^8^ cm^−3^, OH = 6 × 10^7^ cm^−3^, HO_2_ = 1 × 10^9^ cm^−3^, NO = 0.12–0.81 ppbv, NO_2_ = 0.57–0.84 ppbv, RH = 52% and temperature = −48 °C.

In Extended Data Fig. 4, we show two further examples at −50 °C, without (left panels) and with (right panels) NO_*x*_. Both experiments started with acid concentrations below the limit of detection (2 × 10^4^ cm^−3^). Extended Data Fig. 4c shows a threefold enhancement in *J*_1.7_ with the transition from neutral to galactic cosmic ray conditions, reaching 0.7 cm^−3^ s^−1^ at 2.3 × 10^7^ cm^−3^ non-nitrate IP-OOM (IP_0N_) but showing very little further increase at higher (beam) ionization rates. This indicates that ions can enhance the stability of IP-OOM molecular clusters at especially low concentrations of acids or IP-OOM. When NO_*x*_ is present and under galactic cosmic ray conditions (Extended Data Fig. 4d), the initial nucleation rate is 1.7 cm^−3^ s^−1^ at 3.5 × 10^7^ cm^−^^3^ IP_0N_ plus 1.3 × 10^8^ cm^−3^ IP_1-2N_. This comparison suggests that nitrate IP-OOM are less effective for nucleation than non-nitrate IP-OOM. In Extended Data Fig. 4b, sulfuric acid was increased in steps from 2 × 10^4^ to 3 × 10^6^ cm^−3^ and *J*_1.7_ increased from 1.7 to 43 cm^−3^ s^−1^, demonstrating that the synergy between IP-OOM and sulfuric acid also occurs in the presence of NO_*x*_.

## New particle formation rates

**Extended Data Fig. 5:**
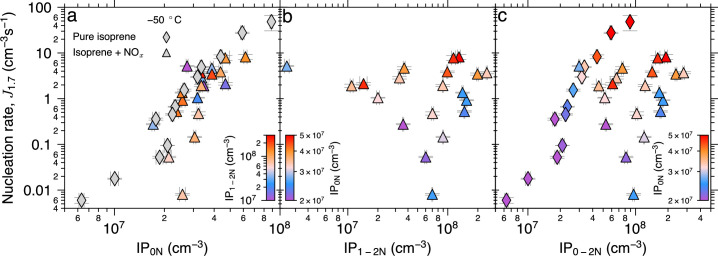
Particle-nucleation rates from IP-OOM at −50 °C, in the absence of acids. Nucleation rates at 1.7 nm, *J*_1.7_, versus IP-OOM without nitrogen, IP_0N_ (**a**), with nitrogen, IP_1N_ + IP_2N_ (**b**) and the sum, IP_0-2N_ (**c**). Panel **a** indicates a mild enhancement of the nucleation rates with increased IP_1N_ + IP_2N_. Panels **b** and **c** confirm that the nucleation rates are only weakly dependent on IP_1N_ + IP_2N_. These data show that non-nitrate IP-OOM are primarily responsible for particle nucleation, with a smaller contribution from nitrate-containing isoprene products. The experimental conditions are: isoprene = 0.04–0.50 ppbv (0.14–1.7 × 10^10^ cm^−3^), O_3_ = 1.2–590 ppbv (4 × 10^10^ to 1.8 × 10^13^ cm^−3^), OH = 0.14–6.40 × 10^7^ cm^−3^, HO_2_ = 1.6–17.0 × 10^8^ cm^−3^, HO_2_/OH ratio = 11–118, NO = 0–0.18 ppbv, NO_2_ = 0–0.74 ppbv, RH = 29–61% and temperature = −50 °C. All experiments are carried out under galactic cosmic ray ionization (ambient-boundary-layer conditions). The error bars represent the standard deviation of the measurement at steady state.

We show in Extended Data Fig. 5 our measurements of *J*_1.7_ versus IP_0N_, IP_1-2N_ and IP_0-2N_ at −50 °C, in the absence of acids (near or below the limit of detection). Extended Data Fig. 5a shows that the nucleation rates increase from around 0.006 cm^−3^ s^−1^ to 48 cm^−3^ s^−1^ by increasing IP_0N_ from 6 × 10^6^ to 9 × 10^7^ cm^−3^. By contrast, there is a relatively weak dependence of the nucleation rate on IP_1-2N_ (Extended Data Fig. 5b,c, which cover a larger IP_1-2N_ range from 2.4 × 10^6^ to 2.5 × 10^8^ cm^−3^). Nevertheless, careful inspection of the data in Extended Data Fig. 5b at the lowest IP_0N_ concentrations does show some dependence of *J*_1.7_ on nitrate IP-OOM. The combined measurements suggest that IP_0N_ are more effective for nucleation than IP_1-2N_, or—equivalently—that nitrate IP-OOM have higher volatilities than non-nitrate IP-OOM. However, at colder temperatures in the upper troposphere, the volatilities of all IP-OOM will decrease and nitrate IP-OOM will contribute more strongly to nucleation.

**Fig. 2:**
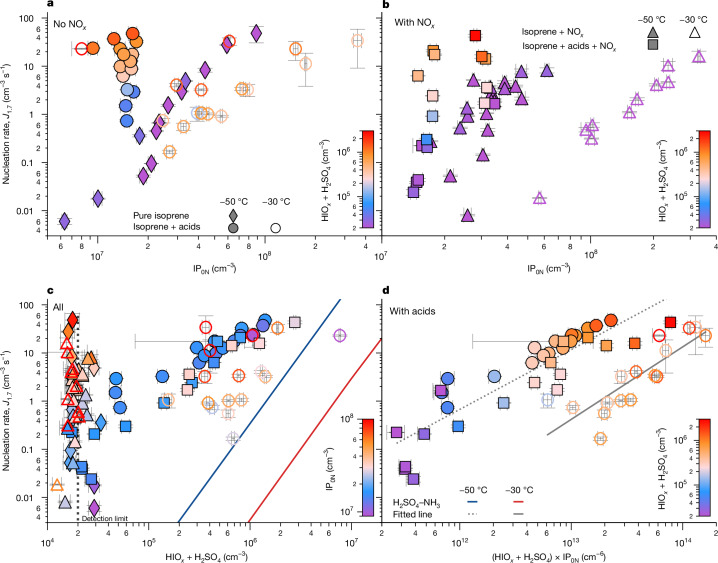
Particle-nucleation rates from IP-OOM at −30 °C and −50 °C, with variable NO_*x*_. **a**–**d**, Nucleation rates at 1.7 nm, *J*_1.7_, versus IP_0N_ without NO_*x*_ present (**a**), IP_0N_ with NO_*x*_ (**b**), HIO_*x*_ + H_2_SO_4_ with and without NO_*x*_ (**c**) and the product (HIO_*x*_ + H_2_SO_4_) × IP_0N_ with and without NO_*x*_ (**d**). Measurements without NO_*x*_ are indicated by diamonds (without acids) and circles (with acids). Measurements with NO_*x*_ are indicated by triangles (without acids) and squares (with acids). Hollow symbols indicate −30 °C and solid symbols indicate −50 °C. In **c**, nucleation rates measured at contaminant acid concentrations are assigned the acid limit of detection (around 2 × 10^4^ cm^−3^). The solid lines in **c** show the nucleation rates expected for H_2_SO_4_ with 4 pptv NH_3_ at −30 °C (red) and −50 °C (blue), both at 60% RH (ref. ^44^). The dashed and solid lines in **d** represent fits to the equation 10^*a*×log10(*x*)+*b*^, in which, for the dashed line, *a* = 1.241 and *b* = −15.065, and for the solid line, *a* = 1.505 and b = −19.948. Panels **a**–**c** show that both IP_0N_ and total acid (HIO_*x*_ + H_2_SO_4_) contribute to the nucleation rate. Panel **d** indicates that it is the product of IP_0N_ and total acid (that is, the dimer formation rate) that best describes the nucleation rate, *J*_1.7_, as the data points cluster into two groups primarily characterized by temperature alone. IP_1-2N_ also contribute to particle nucleation but they are less effective than IP_0N_ (Extended Data Fig. 5). The experimental conditions are: isoprene = 0.04–1.50 ppbv (0.1–4.2 × 10^10^ cm^−3^), O_3_ = 1–590 ppbv (3.7 × 10^10^ to 1.8 × 10^13^ cm^−3^), I_2_ = 0–7.5 × 10^7^ cm^−3^, SO_2_ = 0–4.6 × 10^9^ cm^−3^, OH = 0.11–6.90 × 10^7^ cm^−3^, HO_2_ = 0.6–17.0 × 10^8^ cm^−3^, HO_2_/OH ratio = 11–118, NO = 0–0.22 ppbv, NO_2_ = 0–0.77 ppbv, RH = 29–70% and temperature = −30 °C and −50 °C. The error bars represent the standard deviation of the measurement at steady state. All measurements are made under galactic cosmic ray conditions (natural ionization amounts).

In Fig. 2, we present our measurements of *J*_1.7_ versus vapour concentrations at −30 °C and −50 °C. Here we consider only the IP_0N_ component of IP_0-2N_, following the discussion above. In our experiments, the range of acid concentrations (mostly below 3 × 10^6^ cm^−3^) is representative of the upper free troposphere^47,48^ and the isoprene concentrations (0.14–4.2 × 10^10^ cm^−3^) correspond to those measured in the upper troposphere over tropical rainforests^5^, as do the oxidant concentrations^18^ (OH = 0.11–6.9 × 10^7^ cm^−3^, HO_2_ = 0.6–17 × 10^8^ cm^−3^ and HO_2_/OH ratio = 11–118). These vapour concentrations are sufficient to drive rapid nucleation rates between 0.006 and 48 cm^−3^ s^−1^, which greatly exceed HIO_*x*_ and H_2_SO_4_–NH_3_ nucleation under these conditions^37,44,45^. Such fast nucleation rates can readily account for the high particle-number concentrations of up to 20,000 cm^−3^ observed between 8 and 14 km over the Amazon^2^.

**Extended Data Fig. 6:**
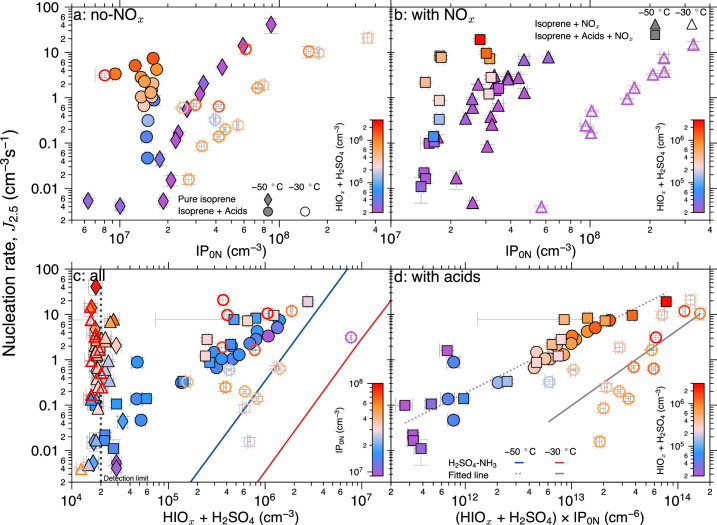
Particle-nucleation rates at 2.5 nm versus vapour concentrations. Nucleation rates at 2.5 nm, *J*_2.5_, versus IP_0N_ (**a**,**b**), HIO_*x*_ + H_2_SO_4_ (**c**) and the product (HIO_*x*_ + H_2_SO_4_) × IP_0N_ (**d**). Panel **a** and **b** show experiments without and with added NO_*x*_. Panel **c** shows all experiments and panel **d** shows experiment with acids, both with and without NO_*x*_. IP_0N_ excludes nitrogen-containing isoprene-oxygenated molecules. The solid lines in panel **c** show the nucleation rates at 1.7 nm expected for H_2_SO_4_ with 4 pptv NH_3_ at 60% RH (ref. ^44^). The dashed and solid lines in panel **d** represent fits to the equation 10^*a*×log10(*x*)+*b*^, in which, for the dashed line, *a* = 1.167 and *b* = −14.743, and for the solid line, *a* = 1.669 and *b* = −22.707. Panels **a** and **b** show that both total acid (HIO_*x*_ + H_2_SO_4_) and IP_0N_ contribute to the nucleation rate. The experimental conditions are: isoprene = 0.04–1.50 ppbv (0.1–4.2 × 10^10^ cm^−3^), O_3_ = 1–590 ppbv (3.7 × 10^10^ to 1.8 × 10^13^ cm^−3^), I_2_ = 0–7.5 × 10^7^ cm^−3^, SO_2_ = 0–4.6 × 10^9^ cm^−3^, OH = 0.11–6.90 × 10^7^ cm^−3^, HO_2_ = 0.6–18.0 × 10^8^ cm^−3^, HO_2_/OH ratio = 11–118, NO = 0–0.22 ppbv, NO_2_ = 0–0.77 ppbv, RH = 29–70% and temperature = −30 °C and −50 °C. All experiments are carried out under galactic cosmic ray ionization (ambient conditions). The error bars represent the standard deviation of the measurement at steady state.

The nucleation rates depend on the concentrations of both IP_0N_ (Fig. 2a,b) and total acid (HIO_*x*_ + H_2_SO_4_; Fig. 2c). Figure  2d suggests that the nucleation rates are linearly dependent on the product of IP_0N_ and total acid concentration, indicating that the critical step is dimer formation of an inorganic acid with a single IP_0N_. We note that the *J*_1.7_ measurements in Fig. 2d at −50 °C with acids and NO_*x*_ (filled square symbols) are systematically around a factor of 2–3 lower than the measurements without NO_*x*_ (filled circles). This is an artefact resulting from a systematic uncertainty in those measurements (filled square symbols), as it is absent when *J*_2.5_ is measured for the same events but with a different particle counter (Extended Data Fig. 6). We measure a 20-fold increase of *J*_1.7_ between −30 °C and −50 °C (Fig. 2d) owing to decreasing IP-OOM volatilities.

## Molecular content of nucleating clusters

**Fig. 3:**
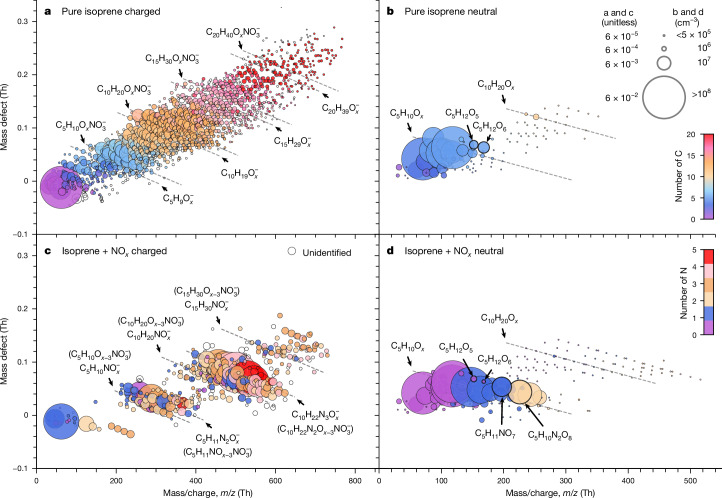
Molecular composition of charged and neutral clusters during IP-OOM nucleation without acids at −50 °C. **a**–**d**, Mass defect (difference from integer mass) versus *m*/*z* during IP-OOM nucleation events without acid and without added NO_*x*_ (**a**,**b**) or with NO_*x*_ (**c**,**d**). The data points are coloured by the number of carbon (**a**,**b**) or nitrogen (**c**,**d**) atoms. The symbol area in **a** and **c** is proportional to the normalized signal intensity by total signal, whereas that in **b** and **d** is proportional to IP-OOM concentrations. The charger ions (Br^−^, NO_3_^−^ and NH_4_^+^) are removed from the molecular formula in **b** and **d**. The data show that IP-OOM—which are around C_5_ for the monomer or C_10_ for the dimer—nucleate at −50 °C. The experimental conditions in **a**–**d** are: isoprene = 0.19, 0.07, 0.28 and 0.07 ppbv, O_3_ = 196.0, 129.0, 1.9 and 1.8 ppbv, OH = 0.3, 0.3, 1.6 and 4.6 × 10^7^ cm^−3^, HO_2_ = 1.8, 2.2, 3.4 and 6.8 × 10^8^ cm^−3^, NO = 0, 0, 0.07 and 0.18 ppbv, NO_2_ = 0, 0, 0.10 and 0.54 ppbv, RH = 31, 57, 66 and 29% and temperature = −49, −49, −48 and −48 °C, respectively. The concentrations of IP_0N_ were unmeasured (instrument was not available), 3.4 × 10^7^, unmeasured and 2.5 × 10^7^ cm^−3^ and the concentrations of IP_1-2N_ were unmeasured, 0, unmeasured and 1.5 × 10^8^ cm^−3^, respectively. The bromide chemical-ionization mass spectrometer was converted to measure charged clusters for the experiments shown in **a** and **c**, under ground-level and upper-tropospheric ion concentrations, respectively.

We have confirmed the nucleation mechanism inferred from Fig. 2d by direct molecular measurements with an Atmospheric Pressure Interface Time-of-Flight (APi-TOF) mass spectrometer during nucleation events without acids at −50 °C (Fig. 3a,c). Negatively charged (ion-induced) nucleation involves sequential accretion of IP-OOM monomers (C_5_ band) or dimers (C_10_ band) to an initial C_*i*_H_*j*_O_*x*_^−^ or NO_3_^−^ ion. When NO_*x*_ is present, a sharp reduction can be seen in the concentration of the C_15_ clusters compared with C_10_ (Fig. 3c). By contrast, the no-NO_*x*_ data (Fig. 3a) show a smooth sequential growth of the clusters out to the detection limit. This indicates that the C_15_ clusters with NO_*x*_ are relatively unstable and have a high evaporation rate back to C_10_ clusters, hence the high concentration of the latter. We infer that the IP_1-2N_ have higher volatility than IP_0N_ and are less effective for nucleation. Nevertheless, C_10_ and C_15_ molecular clusters are seen in Fig. 3c with more than 3N (including NO_3_^−^ core ion), which must include contributions from IP_1-2N_ as well as IP_0N_. This confirms that IP_1-2N_ do indeed participate in ion-induced nucleation, but they are less effective than IP_0N_, which leads to a rate-limiting step from C_10_ to C_15_. This is consistent with the previous conclusions drawn from measurements of nucleation rate.

The composition of neutral clusters and molecules during IP-OOM nucleation without and with NO_*x*_ is shown in Fig. 3b,d, respectively. Here no signal is detected above the C_10_ band owing to the relatively low charging efficiency of the chemical-ionization mass spectrometers, compared with unit efficiency for ions and ion-induced (charged) clusters in the APi-TOF mass spectrometer. Nevertheless, the neutral data reveal a clear shift towards (higher-mass) IP_1N_ and IP_2N_ compounds after the addition of NO_*x*_. Furthermore, comparison with the corresponding charged clusters (Fig. 3a,c) shows that nucleation favours the more highly oxygenated compounds with lower volatility.

**Extended Data Fig. 7:**
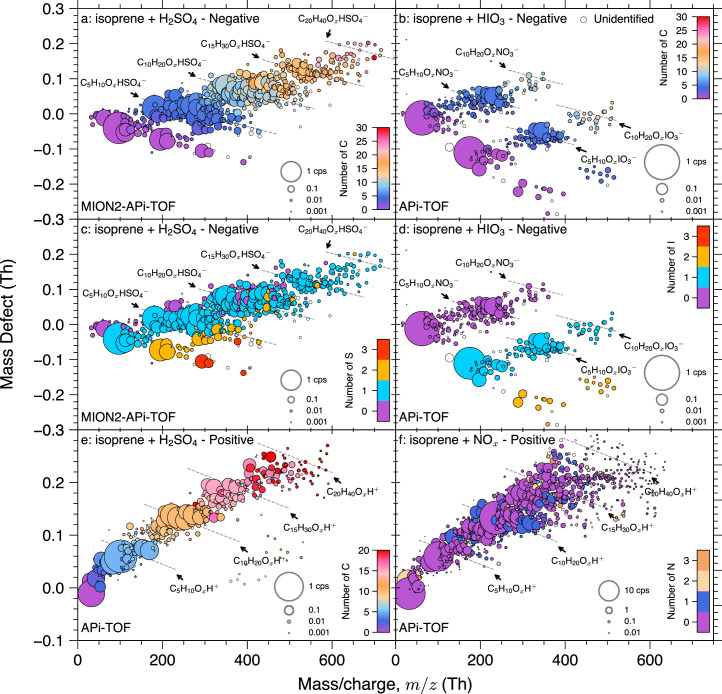
Molecular composition of charged clusters with sulfuric acid, iodic acid and no acid during IP-OOM nucleation at −50 °C. Mass defect (difference from integer mass) versus *m*/*z* (mass-to-charge ratio) during nucleation events for ions and negatively charged (**a**–**d**) and positively charged (**e**,**f**) molecular clusters measured with two APi-TOF instruments (see Methods), coloured by the number of carbon (**a**,**b**,**e**), sulfur (**c**), iodine (**d**) and nitrogen (**f**) atoms. The data are without NO_*x*_ (**a**–**e**) and with NO_*x*_ (**f**). In panel **d**, the (purple) clusters with no iodine contain a NO_3_^−^ ion. The symbol area is proportional to the ion signal rate (cps, counts s^−1^). It should be noted that the APi-TOF used in this experiment is less sensitive than the MION2-APi-TOF and thus the absolute ion signals and cluster population should not be compared between the instruments. Together with Fig. 3, our results show that ion-induced nucleation occurs both with pure IP-OOM and with further H_2_SO_4_ and HIO_*x*_. The experimental conditions for **a**,**c**,**e** are: isoprene = 0.19 ppbv (5.9 × 10^9^ cm^−3^), O_3_ = 93 ppbv (2.9 × 10^12^ cm^−3^), I_2_ = unmeasured, SO_2_ = unmeasured, OH = 3.0 × 10^6^ cm^−3^, HO_2_ = unmeasured, RH = 63% and temperature = −49 °C. The concentrations of IP_0N_ = unmeasured, H_2_SO_4_ = 3.0 × 10^5^ cm^−3^ and HIO_*x*_ = 2.8 × 10^4^ cm^−3^. The experimental conditions for **b**,**d** are: isoprene = 0.09 ppbv (2.8 × 10^9^ cm^−3^), O_3_ = 97 ppbv (3 × 10^12^ cm^−3^), I_2_ = 5.1 × 10^7^ cm^−3^, SO_2_ = 0, OH = 3.4 × 10^6^ cm^−3^, HO_2_ = 7.6 × 10^7^ cm^−3^, RH = 63% and temperature = −49 °C. The concentrations of low-volatility vapours are IP_0N_ = 1.3 × 10^7^ cm^−3^, H_2_SO_4_ = 8.3 × 10^3^ cm^−3^ and HIO_*x*_ = 6.7 × 10^5^ cm^−3^. The experimental conditions for **f** are: isoprene = 0.12 ppbv (3.7 × 10^9^ cm^−3^), O_3_ = 1.2 ppbv (3.7 × 10^10^ cm^−3^), SO_2_ = 1.2 × 10^8^ cm^−3^, OH = 6.2 × 10^7^ cm^−3^, HO_2_ = 1 × 10^9^ cm^−3^, RH = 42%, NO = 0.25 ppbv, NO_2_ = 0.78 ppbv and temperature = −48 °C. The concentrations of low-volatility vapours are IP_0N_ = 1.2 × 10^7^ cm^−3^, H_2_SO_4_ = 2.4 × 10^5^ cm^−3^ and HIO_*x*_ = 0 cm^−3^.

In Extended Data Fig. 7, we show the molecular composition of charged clusters in the presence of trace amounts of sulfuric acid (Extended Data Fig. 7a,c,e) and iodic acid (Extended Data Fig. 7b,d) during IP-OOM nucleation at −50 °C. The data are without NO_*x*_ (Extended Data Fig. 7a–e) and with NO_*x*_ (Extended Data Fig. 7f). These measurements confirm that the same nucleation mechanism occurs after addition of acids as seen previously without acids, that is, sequential addition of IP-OOM monomers (C_5_) or dimers (C_10_) to a core acid ion. Here the negatively charged core ions comprise the pure monomer, dimer or trimer of sulfuric acid (Extended Data Fig. 7a,c) with HSO_4_^−^, or iodic acid with IO_3_^−^ (Extended Data Fig. 7b,d), whereas the positively charged core ions (Extended Data Fig. 7e,f) comprise an IP-OOM^+^. The core acid anion serves only to stabilize the initial molecular cluster with a single IP-OOM; there is no evidence for accretion of further acid molecules as the clusters grow, as expected at these very low acid concentrations (3–7 × 10^5^ cm^−3^).

## Particle growth rates

**Fig. 4:**
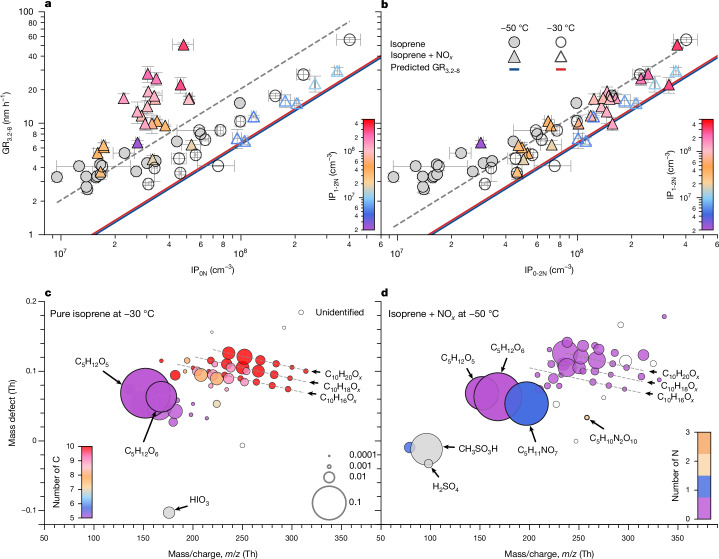
Particle growth rates and particle composition. **a**,**b**, Particle growth rates from 3.2 to 8.0 nm, GR_3.2-8_, versus IP_0N_ (**a**) and IP_0-2N_ (**b**). The solid lines show the predicted GR_3.2-8_ at the kinetic limit assuming monomer condensation and without considering any dipole enhancement. The prediction assumes that IP-OOM have a general formula of C_5_H_12_O_6_ and a density of 1.34 g cm^−3^ (ref. ^50^). The dashed lines are linear fits to the experimental data of the form GR_3.2-8_ = 2.04 × 10^−7^ × IP_0N_ (**a**) and GR_3.2-8_ = 1.23 × 10^−7^ × IP_0-2N_ (**b**). **c**,**d**, Mass-defect plots showing the molecular composition of particles measured with the FIGAERO at −30 °C (Br-FIGAERO) and −50 °C (I-FIGAERO), with particle geometric mean sizes ranging from 6 to 20 nm. The colour legends indicate the number of atoms of carbon (**c**) and nitrogen (**d**). The symbol area is proportional to the normalized signal by the sum of measured signals. The charger ions are removed from the molecular formula. The annotations show the molecular formula of the particle-phase compounds. Our measurements show that both IP_0N_ and IP_1-2N_ drive rapid early particle growth and that C_5_H_12_O_5-6_ are the main condensing vapours in IP_0N_. The experimental conditions in **a** and **b** are: isoprene = 0.05–1.50 ppbv (0.15–4.40 × 10^10^ cm^−3^), O_3_ = 1–592 ppbv (3.6 × 10^10^ to 1.8 × 10^13^ cm^−3^), I_2_ = 0–1.1 × 10^8^ cm^−3^, SO_2_ = 0–4.9 × 10^9^ cm^−3^, OH = 0.09–7.40 × 10^7^ cm^−3^, HO_2_ = 0.6–19.0 × 10^8^ cm^−3^, NO = 0–0.26 ppbv, NO_2_ = 0–0.80 ppbv, RH = 29–72% and temperature = −30 °C and −50 °C. The experimental conditions for in **c** are: isoprene = 1.1 ppbv, O_3_ = 184 ppbv, I_2_ = 2.9 × 10^7^ cm^−3^, SO_2_ = 2.7 × 10^7^ cm^−3^, OH = 2.3 × 10^6^ cm^−3^, HO_2_ = 0.8 × 10^8^ cm^−3^, NO = 0 ppbv, NO_2_ = 0 ppbv, RH = 72%, temperature = −30 °C and DMS = 0 ppbv and those for in **d** are: isoprene = 0.2 ppbv, O_3_ = 1 ppbv, I_2_ = 0, SO_2_ = 1.2 × 10^8^ cm^−3^, OH = 5.2 × 10^7^ cm^−3^, HO_2_ = 1.2 × 10^9^ cm^−3^, NO = 0.11 ppbv, NO_2_ = 0.65 ppbv, RH = 38%, temperature = −48 °C and DMS = 0.16 ppbv. The vertical error bars represent the statistical uncertainty in the appearance-time growth-rate measurements derived from the 95% confidence interval on the growth-rate fit. The horizontal error bars represent the standard deviation of measured IP_0N_ or IP_0-2N_ during the growth period.

Our measurements indicate that IP_0N_ (and, less effectively, IP_1-2N_) are rapidly nucleating together with H_2_SO_4_ and HIO_*x*_ at −30 °C and −50 °C (Fig. 2). We therefore expect that IP-OOM will also drive rapid particle growth at larger sizes as the Kelvin (curvature) barrier falls and progressively higher-volatility compounds are able to condense onto the particles—as previously seen for new particle formation from α-pinene oxidation products^49^. We show in Fig. 4 our measurements of particle growth rates between 3.2 and 8.0 nm versus IP_0N_ (Fig. 4a) and IP_0-2N_ (Fig. 4b) for experiments with and without NO_*x*_ at −30 °C and −50 °C. Figure 4a shows that IP_1-2N_ are strongly contributing to early growth of particles; the measured growth rates cannot be explained by IP_0N_ alone. This is confirmed in Fig. 4b, in which all data (with or without NO_*x*_ and at either temperature) are consistent with having the same dependency of growth rate on IP_0-2N_.

We find that isoprene at upper-tropospheric concentrations will drive particle growth rates between 3 and 56 nm h^−1^. These rapid growth rates imply that new IP-OOM particles can reach several tens of nanometres in size within a few hours, which will help to prevent them from evaporation when descending to lower altitudes and warmer temperatures. Within measurement uncertainties, particle growth rates at all temperatures below −30 °C are linearly dependent on the IP_0-2N_ concentration, and they reach the kinetic limit. As expected from their low concentrations, the growth rates show no correlation with H_2_SO_4_ and HIO_*x*_ (not shown).

We have verified these observations by direct particle-phase measurements made with the Filter Inlet for Gases and AEROsols (FIGAERO; Fig. 4c,d). The main IP_0-2N_ compounds in the particles are two second-generation oxidation products (C_5_H_12_O_5_ and C_5_H_12_O_6_) formed from reactions of ISOPOOH with OH, which together constitute 30% of the total signal. The same two compounds have been previously identified in the particle phase by isoprene experiments at room temperature^30^. The remaining particle-phase compounds are largely C_10_ IP-OOM dimers, in agreement with their measured low volatilities. The non-nitrate C_10_ dimers are suppressed by the presence of NO_*x*_, as seen by comparing Fig. 4c with Fig. 4d. We note that the nitrate IP-OOM in Fig. 4d are probably under-represented because we measured that they have lower evaporation temperatures and so may escape thermal-desorption measurement in the FIGAERO.

## Upper-tropospheric particle formation

**Extended Data Fig. 8:**
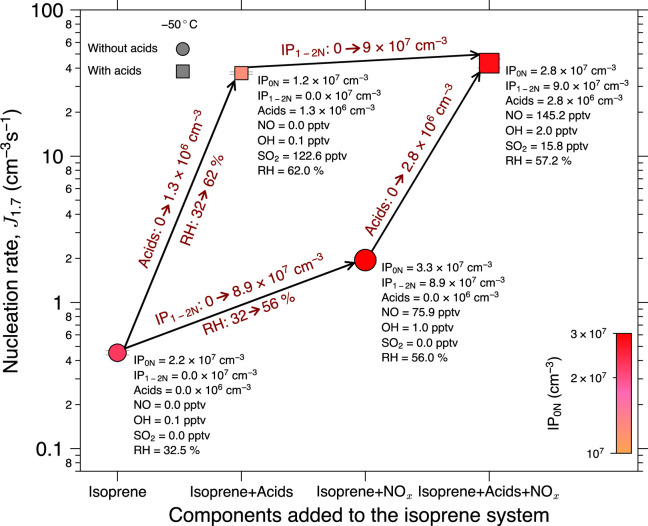
Four experiments that demonstrate the influence of acids and NO_*x*_ on IP-OOM nucleation at −50 °C. Four experiments showing nucleation rates at 1.7 nm, *J*_1.7_, measured at similar IP_0N_ concentrations, 1.2–3.3 × 10^7^ cm^−3^, but with added acids, NO_*x*_ and acids + NO_*x*_, respectively. The marker area is proportional to the IP_0N_ concentration. The experimental conditions are shown next to the data points. Circles correspond to absence of acids and squares include acids. The data show that extremely low H_2_SO_4_ concentrations, 1.3–2.8 × 10^6^ cm^−3^, strongly enhance the nucleation rates regardless of the absence or presence of NO_*x*_. On the other hand, additions of high nitrate IP-OOM concentrations around 10^8^ cm^−3^ only produce relatively small increases in nucleation rates.

In summary, we find that IP-OOM rapidly form new particles at upper-tropospheric concentrations and temperatures below −30 °C. Moreover, the nucleation rates are up to 100 times faster in the presence of extremely low concentrations of sulfuric acid or iodine oxoacids, reaching rates around 30 cm^−3^ s^−1^ at −50 °C and acid concentrations of 10^6^ cm^−3^. In the presence of NO_*x*_, a large fraction of IP-OOM—around 23–88%—are found to contain either one or two nitrogen atoms. We find that nitrate IP-OOM contribute relatively weakly to particle nucleation compared with non-nitrate IP-OOM at the same concentration. However, cooler temperatures will favour nucleation from nitrate isoprene products^13^. Both non-nitrate and nitrate IP-OOM are equally effective at driving rapid particle growth at several tens of nm h^−^^1^, at all temperatures below −30 °C. We present in Extended Data Fig. 8 four nucleation rate measurements that schematically encapsulate the effect of acids and NO_*x*_ on IP-OOM nucleation.

**Fig. 5:**
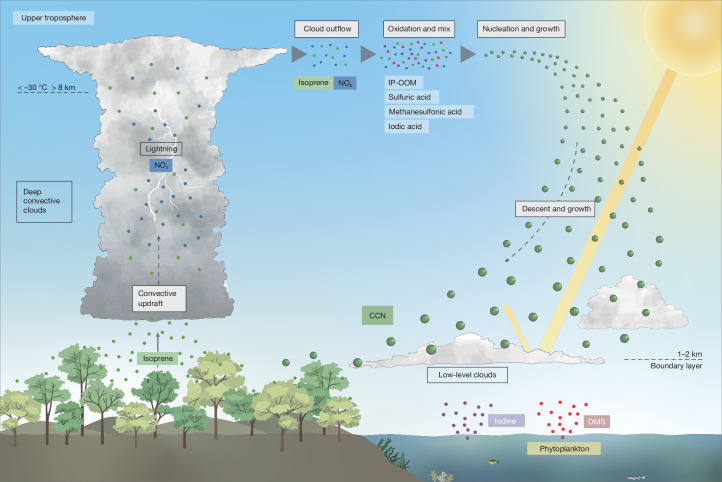
Schematic of new particle formation from isoprene in the upper troposphere. Isoprene from forests is efficiently transported at night by deep convective clouds into the upper troposphere. During daylight, the isoprene accumulated overnight, together with daytime-convected isoprene, reacts with hydroxyl radicals and NO_*x*_ from lightning to produce IP-OOM. The IP-OOM combine with trace ambient acids to produce high particle-number concentrations at cold temperatures below −30 °C. The newly formed particles grow rapidly over several hours to days while following the descending air masses. This mechanism may provide an extensive source of CCN for shallow continental and marine clouds, which strongly influence Earth’s radiative balance.

Our measured nucleation and growth rates provide the mechanistic missing link connecting the presence of abundant isoprene in the tropical upper troposphere^5^ with the high particle-number concentrations found at high altitudes over the Amazon^2^. Our findings reveal a new mechanism (Fig. 5) that switches on rapid particle nucleation in extensive regions of the upper troposphere. Isoprene emitted by tropical rainforests is efficiently transported by deep convective clouds and released at cloud outflows in the upper free troposphere^10,17^. During night-time, high isoprene concentrations build up in the upper troposphere^5^ as a result of relatively slow oxidation by ozone and nitrate radicals^35^. During daytime, the isoprene is rapidly oxidized by hydroxyl radicals and mixed with NO_*x*_ from lightning to produce IP-OOM. These mix with trace ambient acids to drive rapid particle nucleation and growth at cold temperatures below around −30 °C. Peak IP-OOM concentrations—and therefore the fastest new particle formation rates—will occur shortly after sunrise when the isoprene accumulated during the night is oxidized during the first 1–2 h of daylight^5^. However, later in the day, the increase of OH and HO_2_ will accelerate reactions that form acids and favour production of non-nitrate IP-OOM from daytime-convected isoprene, which may lead to further particle nucleation. The newly formed particles grow over periods of several days by further condensation of low-volatility vapours, including acids. Model studies show that particles nucleated in the upper free troposphere over the Amazon are gradually transported downwards on horizontal scales much larger than 1,000 km (ref. ^8^).

Isoprene is the most abundant non-methane hydrocarbon emitted into the atmosphere, but its ability to nucleate particles in the boundary layer is considered negligible. Our findings show, however, that isoprene emitted by forests can drive rapid particle nucleation and growth in the upper troposphere. After further growth and descent to lower altitudes, these particles may represent a globally important source of CCN for shallow continental and marine clouds, and so influence Earth’s radiative balance. Isoprene from forests may therefore provide a major source of biogenic particles in both the present-day and pre-industrial atmospheres that are at present unaccounted in atmospheric chemistry and climate models.

## Methods

### The CLOUD experiments

The CERN CLOUD chamber^36^ was used to conduct the experiments presented in this study. CLOUD is an electropolished, stainless-steel, 26.1-m^3^ chamber designed to study new particle formation under the full range of tropospheric and lower-stratospheric conditions. The thermal housing around the chamber is able to control the temperature from 208 to 373 K with high precision (±0.1 K)^51^. CLOUD was operated at a pressure of approximately 965 ± 5 mbar in this study. To avoid cross-contamination between different experimental programmes and to achieve extremely low NH_3_ concentrations, the chamber is cleaned by rinsing the chamber walls with ultrapure water and heating to 373 K for more than 24 h. To maintain cleanliness and ensure minimal contamination, ultrapure synthetic air—derived from mixing cryogenic liquids (21% oxygen and 79% nitrogen)—is continuously injected into the chamber. The chamber is characterized by a low loss rate, with condensation sink values comparable with those observed in pristine environments.

Various light sources are positioned in the CLOUD chamber to selectively drive photochemistry. OH production is initiated by illuminating O_3_ with a UV fibre-optic system, a combination of four 200-W Hamamatsu Hg-Xe lamps with wavelengths spanning 250 and 450 nm, a krypton fluoride (KrF) excimer UV laser at 248 nm and a 52-W low-pressure mercury lamp centred at 254 nm. As well as O_3_ photolysis, OH radicals are also produced by photochemical production from nitrous acid (HONO) and hydrogen peroxide (H_2_O_2_). Both the HONO and H_2_O_2_ generators were designed specifically for CLOUD experiments. Following the same principle as an earlier study^52^, a gas–liquid mixture of HONO is synthesized from continuous mixing of H_2_SO_4_ (Sigma Aldrich, 99%) with sodium nitrite (NaNO_2_, Sigma Aldrich, 99%) in a stainless-steel reactor^53^. HONO is transferred from liquid phase to gas phase by flowing nitrogen gas (1–2 l min^−1^) through the reactor. HONO is then introduced into the CLOUD chamber and photolysed by a UV light source centred at 385 nm to produce OH radicals and NO. The HONO reactor is continuously cooled to 5 °C and a cryo-trap is placed between the reactor and the chamber to remove excess water vapour and avoid ice blockage of the chamber input pipe. Gaseous H_2_O_2_ is produced from bubbling N_2_ gas through a H_2_O_2_ solution. The H_2_O_2_ solution is stored in a glass beaker contained in a stainless-steel container at a constant temperature of 5 °C. A different combination of UV sources is used to photolyse H_2_O_2_ to produce different amounts of OH radicals.

A green light sabre centred at 528 nm is used to photolyse molecular iodine (I_2_). All light systems are continuously monitored by a spectrometer and an array of photodiodes at the bottom of the chamber. Dedicated actinometry experiments allow quantitative determination of actinic fluxes of the light system at different intensities.

Particle formation under different ionization regimes is simulated by combining a strong electric field (±30 kV) and the pion beam produced by the CERN Proton Synchrotron. The electric field eliminates natural ions in less than 1 s, thus creating ion-free conditions (neutral experiments). The pion beam produced by the CERN Proton Synchrotron enhances ion production on top of the galactic cosmic rays. Two magnetically coupled stainless-steel fans mounted at the top and bottom of the chamber enable uniform spatial mixing of particles and vapours within a few minutes. Iodine is injected into the chamber from a temperature-controlled evaporator containing crystalline iodine (I_2_, Sigma-Aldrich, 99.999% purity) at the bottom of the chamber. The SO_2_ (Carbagas, 100 parts per million by volume (ppmv) in N_2_) and isoprene (PanGas, 1,000 ppmv in N_2_) are injected into the chamber from pressurized gas cylinders and the O_3_ is introduced to the chamber by passing O_2_ through an ozone generator.

The data presented in this study were collected in two consecutive CLOUD campaigns (CLOUD15 and CLOUD16). The CLOUD15 and CLOUD16 campaigns were carried out from September to November in 2022 and 2023, respectively. Because the experiments reported in this study were carried out at extremely low temperatures (−30 °C and −50 °C), heat-insulation systems (CLOUD15) and active cooling systems (CLOUD16) were used to reduce measurement systematic error. The heat-insulation systems were primarily made with thermal insulation foam to isolate the instrument inlet system from ambient air. The active cooling systems involved circulating the air inside the chamber thermal housing, at the same temperature as the chamber, around the inlet systems of different instruments. The active cooling systems were also wrapped with thermal insulation foam to allow for more effective inlet cooling. These cooling systems were applied to all mass spectrometers and particle counters, except a butanol condensation particle chamber (CPC; TSI 3776), a nano-scanning mobility particle sizer (nano-SMPS, TSI 3938) and a long-SMPS (TSI 3082), which used a heat-insulation system in both campaigns to act as a standard to avoid systematic errors resulting from changing from the heat-insulation system to the active cooling system.

### Measurement of chemical composition

#### Ozone (O_3_)

O_3_ was monitored using a gas monitor (Thermo Environmental Instruments, TEI 49C).

#### Hydroxyl radicals (OH)

The OH radical was measured by HORUS^54^ (HydrOxyl Radical measurement Unit based on fluorescence Spectroscopy).

#### Hydroperoxyl radical (HO_2_)

The HO_2_ radical was primarily measured using the bromide chemical-ionization mass spectrometer coupled with a multi-scheme chemical-ionization inlet-2 (Br-MION2-CIMS)^55^ and HORUS in both CLOUD15 and CLOUD16 campaigns. HORUS measures HO_2_ by chemically converting it to OH by NO. However, the RO_2_ radical (organic peroxy radicals) produced from isoprene oxidation may also contribute to the HO_2_ signal measured by HORUS, as the reaction between RO_2_ + NO can also produce OH radicals. By contrast, the HO_2_ measurement by Br-MION2-CIMS is less ambiguous, as it is defined by the peak HO_2_Br^−^ (ref. ^55^). However, the measurement of HO_2_ by Br-MION2-CIMS is severely affected by air–water content^55^, making offline calibration difficult. Therefore, the HO_2_ measurement by Br-MION2-CIMS was calibrated by HORUS under RO_2_ radical-free conditions. The online calibration was carried out for every absolute humidity condition reported in this manuscript. During a small section in which the primary ions of Br-MION2-CIMS were saturated by either HONO or H_2_O_2_, either the low-pressure bromide chemical-ionization mass spectrometer or HORUS was used to complement the HO_2_ measurement after intercomparing the data with Br-MION2-CIMS and HORUS during experiments without HONO and H_2_O_2_. The precision of OH and HO_2_ data acquired by the HORUS instrument is quantified at 13% and 7%, respectively, with uncertainties calculated at 1*σ* over a 10-min averaging period. Furthermore, the systematic error of the measurement is calculated to be 12% for OH and 30% for HO_2_.

#### Nitrogen oxide (NO) and dioxide (NO_2_)

NO was measured by detecting the chemiluminescence of NO and O_3_ using a chemiluminescence detector (ECO PHYSICS, CLD 780TR). This instrument was calibrated by a second NO monitor (ECO PHYSICS, CLD 780TR), which—in turn—was calibrated using the CMK5 Touch dilution system (Umwelttechnik MCZ GmbH) with a NO bottle (Praxair, 1.00 ppmv in N_2_) and synthetic air (Nippon Gases, hydrocarbon-free). The first detector, which provides data for this study, was found to contain background values that have been subtracted in this study. NO_2_ was measured by a cavity-attenuated phase-shift nitrogen dioxide monitor (CAPS NO_2_, Aerodyne Research Inc.). Hourly, the instrument undergoes a 5-min background measurement of pure N_2_ gas. During the 5-min background measurements, data have been interpolated to give a continuous time series. The NO_2_ monitor was calibrated using a custom-made cavity-enhanced differential optical absorption spectroscopy instrument^56^. After the subtraction of an average instrument background concentration, the final NO_2_ concentration was obtained.

#### Nitrous acid (HONO) and hydrogen peroxide (H_2_O_2_)

Both HONO and H_2_O_2_ were detected using bromide chemical-ionization mass spectrometry^55^, as they exhibit reasonable affinity with the bromide anion. Direct calibrations of these two species were not carried out on-site and the current estimation assumes that they share the same detection sensitivity as H_2_SO_4_ (a low-limit estimation). Because these species serve as the precursors of OH and NO radicals, which were reliably traced, the concentrations of HONO and H_2_O_2_ are not crucial to the reported results and are therefore omitted from this study.

Two bromide chemical-ionization systems were used to detect HONO and H_2_O_2_. The first system, Br-MION2-CIMS, offers sensitive detection of both species at concentrations below about 10^10^ cm^−^^3^, with a detection limit of around 6 × 10^6^ cm^−^^3^ (H_2_O_2_) and 1.6 × 10^5^ cm^−^^3^ (HONO). However, in some experiments, the estimated HONO and H_2_O_2_ concentrations exceeded 10^10^ cm^−^^3^. The second system, Br-AIM-CIMS, uses bromide chemical-ionization at low pressure in combination with an active water feedback loop to control the Br-hydration in the ion molecule reactor and avoids saturation. Br-AIM-CIMS was used to measure concentrations from above the detection limit of 4.8 × 10^7^ cm^−^^3^ (HONO) and 3.3 × 10^7^ cm^−3^ (H_2_O_2_), based on a calibration factor of 3 × 10^12^ for HONO and H_2_O_2_.

#### Sulfur dioxide (SO_2_)

To measure the concentration of SO_2_, a gas monitor (Thermo Fisher Scientific Model 42i-TLE) was used. However, as the SO_2_ concentrations in our experiments were usually below 5 × 10^9^ cm^−3^ (150 pptv), we also used the Br-MION2-CIMS to measure SO_2_ (ref. ^55^) in both CLOUD15 and CLOUD16 campaigns. The measurement of SO_2_ by Br-MION2-CIMS is substantially affected by air–water content, so we conducted online SO_2_ calibration using the SO_2_ monitor at both −30 °C and −50 °C. The derived calibration factors are 1.7 × 10^13^ at −30 °C and 1.5 × 10^11^ at −50 °C for CLOUD15 and 3.1 × 10^11^ at −50 °C for CLOUD16. During the experiments, when the primary ions of Br-MION2-CIMS were saturated by either HONO or H_2_O_2_, the Br-AIM-CIMS was used to complement the SO_2_ measurement. With an active water sensitivity control, Br-AIM-CIMS measures SO_2_ concentrations from above the detection limit of 3 × 10^7^ cm^−^^3^ with a constant calibration factor of 20 × 10^12^ at −30 °C and −50 °C.

#### Sulfuric acid (H_2_SO_4_)

To ensure the quality of the reported data, we monitored H_2_SO_4_ concentrations using two chemical-ionization mass spectrometers: the nitrate chemical-ionization mass spectrometer (NO_3_-CIMS) and the MION2-CIMS operating in bromide chemical-ionization mode (Br-MION2-CIMS^55^). Furthermore, isotopically labelled H^15^NO_3_ was used during the CLOUD16 campaign to distinguish the nitrogen atom originating from the analyte with the reagent ion. The H_2_SO_4_ calibration was carried out by two independent calibration systems. The first set-up used the original calibration box designed by Kürten et al.^57^ along with their in-house calibration scripts. The second set-up is similar to the original version but with different physical dimensions. Also, the recently developed open-source MARFORCE model is used to simulate H_2_SO_4_ production in both calibration set-ups^55^.

In total, we conducted seven calibration experiments at different stages of the CLOUD15 campaign, and each CIMS instrument was calibrated using both calibration set-ups. Two calibrations were performed for the Br-MION2-CIMS, resulting in equivalent H_2_SO_4_ calibration factors of 157% and 149%. For the NO_3_-CIMS, five calibrations were carried out, resulting in equivalent calibration factors of 88%, 100%, 95%, 154% and 164%. Given that the NO_3_-CIMS provided most of the H_2_SO_4_ concentration in this study, we use the calibration carried out immediately after the experiments for this study. This results in a calibration factor of 6.2 × 10^9^ cm^−3^ for the NO_3_-CIMS and an equivalent calibration factor of 9.0 × 10^9^ cm^−3^ for the Br-MION2-CIMS. We use the minimum and maximum of the seven calibrations, ranging from 88% to 164%, as the systematic error of the H_2_SO_4_ detection for CLOUD15. It is important to note that we had to change the optimal inlet flow rates of the Br-MION2-CIMS at −30 °C and −50 °C. The varying temperatures and flow rates result in different inlet loss rates, all of which have been accounted for in this dataset.

As well as the normal H_2_SO_4_ calibration, we conducted a set of iodine oxoacid nucleation experiments at −10 °C, similar to those presented in ref. ^37^. The nucleation rates in these experiments are comparable with all of our earlier experiments, further enhancing our confidence in the reported acid concentrations.

In the CLOUD16 campaign, a total of seven calibration experiments were carried out. Two calibration experiments were conducted for the Br-MION2-CIMS, before and after the presented experiments. The results yield equivalent H_2_SO_4_ calibration factors of 120% and 118%. For the labelled NO_3_-CIMS, six calibrations were performed in total, three before the isoprene experiments, resulting in equivalent calibration factors of 100%, 99% and 88%. It is important to note that, during the last few days of the isoprene experiments, the NO_3_-CIMS suffered from a pump failure that may have caused a shift (by up to 20%) in the calibration factor owing to a slight change in the sample flow. This potentially affects only two experiments in this study. To correct for this, we have assumed a linear correlation between the sample flow and calibration factor. The failing pumps were then replaced and the data from the rest of the experiments were calibrated after the presented experiments, with two calibrations that yielded equivalent calibration factors of 190% and 185%. This yields a calibration factor of 1 × 10^10^ cm^−3^ for the labelled NO_3_-CIMS and an equivalent calibration factor of 1.9 × 10^10^ cm^−3^ for the Br-MION2-CIMS. By considering all of the calibration experiments, the systematic error of H_2_SO_4_ detection for CLOUD16 is estimated to range from 88% to 120%. Furthermore, using these two instruments, after applying their respective calibration factors, we compared the measured methanesulfonic acid concentrations from the CLOUD chamber at −50 °C. This comparison demonstrated a good agreement, confirming the accuracy of the calibrations.

#### Iodine species

We measured iodic acid (HIO_3_) and iodous acid (HIO_2_) using both the NO_3_-CIMS and Br-MION2-CIMS and we use the same calibration factor as H_2_SO_4_ in the data analysis, similar to our earlier studies^37,45,55,58,59^. We used Br-MION2-CIMS to measure I_2_, which is detected at the collision limit, shown by our recent studies^55,60^.

#### Isoprene

Isoprene was measured by a proton transfer reaction mass spectrometer using the hydronium chemical-ionization method^61^ (H_3_O-PTR-MS). This particular instrument used in this study is an adapted version, which is explained in greater detail previously^62^.

#### ISOPOOH and IEPOX detection and separation

Measuring and distinguishing between ISOPOOH and IEPOX can be experimentally challenging owing to their identical molecular formula (C_5_H_10_O_3_). As a result, mass-spectrometric methods often detect them together at the same exact mass in the same peak^35^. To address this issue, techniques such as tandem mass spectrometry have been used to separate ISOPOOH and IEPOX from each other^29^.

In this study, these two isomeric compounds were measured both by the Br-MION2-CIMS and the proton transfer reaction mass spectrometer 3 (ref. ^63^) operating in ammonium chemical-ionization mode (NH_4_-PTR3-CIMS^64^). NH_4_-PTR3-CIMS measured ISOPOOH and IEPOX primarily as clusters with ammonium cation, as the proton affinity (see the ‘Quantum-chemical calculations’ section) of NH_3_ (204.25 kcal mol^−1^) is higher than that of 1,2-ISOPOOH (198.31 kcal mol^−1^), 4,3-ISOPOOH (195.51 kcal mol^−1^) and cis-β-IEPOX (204.11 kcal mol^−1^). In this study, we also aim to investigate the capability of the Br-MION2-CIMS in detecting ISOPOOH and IEPOX. We calculate the formation free enthalpies of 1,2-ISOPOOH (−27.5 kcal mol^−1^), 4,3-ISOPOOH (−26.9 kcal mol^−1^) and cis-β-IEPOX (−28.0 kcal mol^−1^) with the bromide anion, respectively. We find that the formation free enthalpies are almost equal to the value of hypoiodous acid (HOI) clustered with the bromide anion (26.9 kcal mol^−1^), as presented in ref. ^55^. Because the instrument used in ref. ^55^ and in this study is the same and the instrument tuning is identical, the fragmentation of these bromide anion cluster ions should be comparable. He et al.^55^ calibrated both the H_2_SO_4_ and the HOI, and the calibration factor of HOI was approximately two times larger than that of H_2_SO_4_. Therefore, the calibration factor used for C_5_H_10_O_3_ is two times the calibration factor for H_2_SO_4_ in this study.

As neither the NH_4_-PTR3-CIMS nor the Br-MION2-CIMS are able to distinguish between ISOPOOH and IEPOX, the reported C_5_H_10_O_3_ in this study is the sum of ISOPOOH and IEPOX. Earlier studies have shown that ISOPOOH is effectively lost to metal surfaces by converting it to methyl vinyl ketone (MVK) and methacrolein (MACR)^42,65,66^, whereas IEPOX is not affected by metal surfaces^67^. However, as the experiments in this study focus on extremely low temperatures (−30 °C and −50 °C), the chamber wall itself may also serve as a cryo-trap^68^ for both ISOPOOH and IEPOX. Therefore, it prevents us from using wall-loss-rate perturbation experiments to separate these two species at these temperatures.

To understand the distribution of ISOPOOH and IEPOX in C_5_H_10_O_3_, we carry out a kinetic simulation using the reduced isoprene oxidation mechanism provided in ref. ^35^. The results are presented in Extended Data Fig. 1b. The simulation is carried out by the F0AM model^43^. The model requires input parameters such as isoprene, OH, HO_2_ and O_3_ concentrations measured by our instruments.

Another important parameter is the wall-loss rate of IP-OOM. We present an experiment in which we manipulate the loss rate of IP-OOM by turning off the light source and increasing the mixing fan spinning rate from 12% to 100% from the equilibrium conditions in Extended Data Fig. 1a. By turning off the light source, the production of IP-OOM stops. Furthermore, by increasing the fan speed, we increase the maximum wall-loss rate from approximately 1.6 × 10^−3^ s^−1^ to 8.5 × 10^−3^ s^−1^. The decay rates of C_5_H_10_O_3_ and C_5_H_12_O_6_, with lifetimes of 137 s and 112 s, respectively, are similar to the decay rate of HIO_3_ (129 s) and also, from previous measurements, H_2_SO_4_. Because HIO_3_ has an accommodation coefficient of unity to the chamber wall, we conclude that C_5_H_10_O_3_ and other species with lower volatilities have similar wall-loss rates. In this study, we apply a general wall-loss rate for these species of 1.6 × 10^−3^ s^−1^. This wall-loss rate is calculated from the measured H_2_SO_4_ wall-loss rate by correcting the diffusivity of C_5_H_12_O_6_ at −30 °C using the method described by our earlier study^58^.

We further conduct simulations for all of our experiments using the same procedure, and the ratio of IEPOX in C_5_H_10_O_3_ versus OH concentration is presented in Extended Data Fig. 1b. As anticipated, the IEPOX ratio is positively correlated with OH concentrations. For further analysis, a fit with an expression of ratio of ${10}^{(0.58\times {\log }_{10}([{\rm{OH}}])-4.6)}$ is plotted.

#### Gas-phase oxidized isoprene products

The gas-phase measurement of IP-OOM was achieved by using a combination of NO_3_-CIMS, Br-MION2-CIMS and NH_4_-PTR3-CIMS. As defined in this study, only the species with carbon and oxygen numbers equal to or larger than 4 are considered in the IP_0-2N_, which are primarily produced from OH oxidation of ISOPOOH and IEPOX with and without involving nitrogen oxides. Furthermore, the particle-phase IP_0-2N_ were monitored by a FIGAERO^69^, which operates with the bromide chemical-ionization method^60^ in CLOUD15 (Br-FIGAERO-CIMS) and with the iodide chemical-ionization method in CLOUD16 (I-FIGAERO-CIMS). These chemical-ionization methods exhibit varying preferences for analytes. For example, the NO_3_-CIMS is renowned for detecting highly oxygenated organic molecules^70^ that contain more than 5 oxygen atoms. The H_3_O-PTR-MS is the only one that can detect isoprene, whereas both the NH_4_-PTR3-CIMS and the Br-MION2-CIMS are capable of detecting semi-volatile organic compounds. Consequently, the combination of these CIMS methods enables the measurement of IP-OOM at different oxidation states.

It is worth mentioning our specialized approach to measuring IP-OOM using Br-MION2-CIMS during experiments involving excess HONO and/or H_2_O_2_, as described previously. In these experiments, the primary ions (Br^−^ and H_2_OBr^−^) were substantially transformed into product ions such as HONOBr^−^, H_2_O_2_Br^−^ and (H_2_O_2_)_2_Br^−^. Consequently, the measurement of IP-OOM could be compromised if HONO and H_2_O_2_ strongly bind with Br^−^, thereby impeding the ligand exchange with IP-OOM. Therefore, we extensively compared the Br-MION2-CIMS measurements with those of NO_3_-CIMS and NH_4_-PTR3-CIMS during experiments with and without such primary ion saturation to ensure reliable measurements. We found that the Br-MION2-CIMS measurement remained uncompromised when we included HONOBr^−^, H_2_O_2_Br^−^ and (H_2_O_2_)_2_Br^−^ as the primary ions. This is probably because of the relatively weak bonding of HONO and H_2_O_2_ with Br^−^, which enables effective charging of IP-OOM by allowing ligand exchange reaction. Quantum-chemical calculations further suggest that the formation free enthalpies of HONOBr^−^ and H_2_O_2_Br^−^ are −23.6 and −21.2 kcal mol^−1^, respectively. These numbers are sufficiently lower than other molecules that are detected at the collision limit by Br-MION2-CIMS^55^.

To produce IP_0-2N_, we conducted a set of experiments in which we varied the concentrations of isoprene (ranging from 1.4 × 10^9^ to 4.2 × 10^10^ cm^−3^) and OH (ranging from 0.1 to 6.9 × 10^7^ cm^−3^) to alter the distribution of oxidation products^35^. To analyse the results of these experiments, we present a generic algorithm to calculate the total sum of gaseous IP_0-2N_ produced, with a focus on those with carbon and oxygen numbers greater than 3:

1. IP-OOM are independently identified by each of the CIMS instruments. Their responses to the isoprene oxidation in the chamber are observed to distinguish them from any background contaminations originating from either the chamber or the ion sources. If an individual IP_0-2N_ is affected by contaminants of the same molecular formula, its background, derived from the nearest cleaning stage, is subtracted from its concentrations.
2. If an IP_0-2N_ is detected by only one of the three CIMS, it is added to the total sum directly.
3. If several CIMS detect species with the same molecular formula, their measured signals are compared in pairs to derive a correlation coefficient. A pair is considered to measure identical molecules if the correlation coefficient is greater than 0.5. However, owing to the transfer of the H_2_SO_4_ calibration factors to the measured IP_0-2N_ (NO_3_-CIMS and Br-MION2-CIMS), the concentration of any molecule with a lower detection efficiency than H_2_SO_4_ may be underestimated. The extent of this underestimation depends on the chemical-ionization method used, as the binding enthalpies of the analyte-Br^−^, analyte-NO_3_^−^ and analyte-NH_4_^+^ may differ. To address this, we add the highest measured concentration of the three CIMS to the IP_0-2N_ and discard the rest, as the highest concentration is probably the closest to the actual concentration.
4. If the correlation coefficient is less than 0.5, we consider that this pair represents two different molecular structures, that is, two isomers or conformers. In this case, both will be added to the IP_0-2N_.

However, maintaining all three instruments to be operational throughout all experiments presents a challenge, for instance, the Br-MION2-CIMS operated in the APi-TOF mode to measure charged clusters. Therefore, we excluded data collected during periods in which any one of the instruments was not available.

#### Charged clusters

Naturally charged clusters were measured with two APi-TOF mass spectrometers (Aerodyne Research Inc.) operating at negative and positive ion mode^71^. The first instrument was equipped with a MION2 operating in the APi-TOF mode (MION2-APi-TOF)^55,72^ by deactivating the inlet voltages responsible for directing charged reagent ions into the sample flow. The second device was coupled with an ion-molecule reaction chamber (APi-TOF). Overall, the APi-TOF was less sensitive than the MION2-APi-TOF. The charged clusters reported in Fig. 3 were measured with the MION2-APi-TOF, which was validated by the APi-TOF. Because the MION2 inlet was operated in bromide chemical-ionization mode in some experiments, part of the data reported in Extended Data Fig. 7 was measured by the APi-TOF.

#### Particle-phase measurements

We measured the chemical composition of small particles using a FIGAERO coupled to a chemical-ionization mass spectrometer^69^. Particles were sampled from the CLOUD chamber onto a 5-µm-pore polytetrafluoroethylene (PTFE) filter (MilliporeSigma). Filter mass loading is dependent on particle distribution in the chamber, collection flow rate (typically 7–8 l min^−1^) and total collection time (1–2 h in this study). After particle collection, the filter was automatically moved to in front of the ion molecule reactor. The filter aligned with a sealed port that constantly flushes pure N_2_. In CLOUD15, the flow rate during chemical measurement was 3 l min^−^^1^ and it was increased to 5 l min^−^^1^ in CLOUD16 for more efficient heat transfer in a longer port. Pure N_2_ was heated from room temperature up to 180 °C using programmed thermal desorption controlled by eyeon software v2.1.4.5. As the filter temperature increased, we detected lower-volatility molecules partitioning back into the gas phase. For the particle filter loadings in this study, we observed that all signals decreased back to the baseline by the end of the heating cycle, indicating no notable remaining mass.

Typically, FIGAERO-CIMS is operated using I^−^ chemical-ionization in a reduced-pressure ion molecule reactor (about 120–150 mbar). Pure N_2_ is flowed around a CH_3_I permeation tube (Vici) and through a 210Po ionizer (NRD LLC) to produce iodide ions. These polarizable ions effectively form adducts with oxygenated organic compounds, with a small fraction of interactions leading to charge transfer between the ion and neutral compound. In CLOUD15, we used Br^−^ chemical-ionization to distinguish between our chemical-ionization reagent and iodine species inside the CLOUD chamber. The set-up is the same as iodide ionization mode except we exchange a CH_2_Br_2_ permeation tube and heat it to 40 °C to increase permeation rates. These chemical-ionization techniques are both sensitive to oxygenated organic compounds, organics with nitrate and sulfate functional groups and inorganic acids^60,69^. Compounds chemically transformed through deprotonation or thermal decomposition have been excluded, as their parent molecule is unknown.

#### Particle number size distribution

The Neutral cluster and Air Ion Spectrometer^73,74^ (NAIS) was used to measure the naturally charged particle number size distribution from 0.8 to 41 nm and the particle number size distribution (naturally charged + neutral) from 2 to 42 nm in both negative and positive polarities. The nano-condensation nucleus counter was used to measure the particle number size distribution between 1 and 3 nm. It consists of a particle size magnifier^75^ (PSM, Airmodus Oy). The PSM, which is an aerosol pre-conditioner, uses diethylene glycol to grow aerosol particles as small as 1 nm to sizes that can be easily detected by a CPC^75^. Furthermore, a butanol CPC (TSI 3776) was used to measure the total number concentration of particles with diameters greater than 2.5 nm. A nano-scanning mobility particle sizer (TSI 3938)^76^ coupled to a butanol CPC (TSI 3776), was used to measure the particle-size distribution within the range 6–65 nm, whereas particles larger than 65 nm were measured using a commercially available long-SMPS (TSI 3082) coupled to a water butanol CPC (TSI 3775).

### Yield of IP_0N_ from ISOPOOH

As shown in a previous section, the IP_0N_ in this study is defined as species with C,O ≥ 4. Therefore, ISOPOOH and IEPOX are not included in the IP_0N_. ISOPOOH and IEPOX are treated as the direct precursors of IP_0N_, which in turn contribute to isoprene new particle formation. It is worth noting that both ISOPOOH and IEPOX undergo oxidation, producing compounds with C,O ≥ 4. However, the reaction rate of ISOPOOH is approximately ten times larger than IEPOX^35^. To account for the difference in reaction-rate coefficients, we predict the ratio of IEPOX in C_5_H_10_O_3_ using the data shown in Extended Data Fig. 1b based on the OH concentrations. Assuming that the concentration of IP_0N_ is at equilibrium, the primary mechanism for IP_0N_ loss is wall deposition, which is approximately equal to the production of IP_0N_ from ISOPOOH and IEPOX. Therefore,

$$\begin{array}{l}[{{\rm{IP}}}_{0{\rm{N}}}]\times {k}_{{\rm{wall}}}\,=\,R\times ({k}_{{\rm{OH}} \mbox{-} {\rm{ISOPOOH}}}\times [{\rm{OH}}]\times [{\rm{ISOPOOH}}]\\ \,\,\,\,\,\,+{k}_{{\rm{OH}} \mbox{-} {\rm{IEPOX}}}\times [{\rm{OH}}]\times [{\rm{IEPOX}}])\end{array}$$

in which *k*_OH-ISOPOOH_ and *k*_OH-IEPOX_ are the reaction-rate coefficients of ISOPOOH (10^−10^ cm^3^ s^−1^) and IEPOX (10^−11^ cm^3^ s^−1^) with OH (ref. ^35^), respectively; [IP_0N_], [OH], [ISOPOOH] and [IEPOX] show concentrations and *k*_wall_ is the wall-loss rate of C_5_H_12_O_6_; *R* represents the yield of IP_0N_ from C_5_H_10_O_3_.

We then define the reacted C_5_H_10_O_3_ (cm^−3^) as:

$${\rm{Reacted}}\,{{\rm{C}}}_{5}{{\rm{H}}}_{10}{{\rm{O}}}_{3}=\frac{{k}_{\text{OH-ISOPOOH}}\times [{\rm{OH}}]\times [{\rm{ISOPOOH}}]+{k}_{\text{OH-IEPOX}}\times [{\rm{OH}}]\times [{\rm{IEPOX}}]}{{k}_{{\rm{wall}}}}$$

The yield of IP_0N_ from reacted C_5_H_10_O_3_ is depicted in Extended Data Fig. 2. We find that the yields of IP_0N_ are approximately 46% at −30 °C and 55% at −50 °C. However, it is essential to note that the detection of C_5_H_10_O_3_, IP_0N_ and OH has various uncertainties. We estimate that the derived yield has an uncertainty of at least a factor of two, with the quantification of IP_0N_ being the main source of uncertainty.

One further source of error in determining the yield is the contribution of highly oxygenated molecule production from the first-generation isoprene hydroxy peroxy radical (ISOPOO, C_5_H_9_O_3_) through auto-oxidation or dimer formation. For example, the reaction between two ISOPOO radicals can generate C_10_H_18_O_4_, and intramolecular H-shift followed by HO_2_ termination of ISOPOO produces C_5_H_10_O_5_. Although these two molecules only contribute to a small fraction of IP_0N_ in this study, other similar channels may contribute to a greater extent to IP_0N_, thereby reducing the yield of IP_0N_ from C_5_H_10_O_3_. As disentangling first-generation and second-generation highly oxygenated molecules from isoprene oxidation is not the objective of this study, future research is necessary to investigate this direction.

### Quantum-chemical calculations

Quantum-chemical methods are used to compute cluster formation free enthalpies and proton affinities. Initially, the Spartan’18 program is used for the conformational sampling with the MMFF method. Subsequently, density function theory (DFT) methods are used to optimize the molecules first at the B3LYP/6-31+G(d) level of theory, followed by optimization and frequency calculations at the ωB97X-D/aug-cc-pVTZ-PP level of theory^77,78^ on conformers within 2 kcal mol^−^^1^ in relative electronic energies. Bromine pseudopotential definitions are obtained from the Environmental Molecular Sciences Laboratory (EMSL) basis set library^79,80^. The DFT calculations are carried out using the Gaussian 16 program^81^. To refine the DFT-calculated enthalpies, an extra coupled-cluster single-point energy correction is performed at the DLPNO-CCSD(T)/def2-QZVPP level of theory on the lowest-energy conformers. This coupled-cluster calculation is conducted using the ORCA program version 5.0.3 (ref. ^82^).

### Calculation of the nucleation and growth rates

The nucleation rate, *J*_1.7_, is calculated on the basis of PSM measurement of particles at a mobility diameter of 1.7 nm (1.4 nm in physical diameter^83^), which are generally considered to be larger than their critical cluster sizes and thus stable.

To determine the nucleation rates, the time evolution of the particle concentration is analysed, taking into account various loss processes that also affect the concentration. However, because loss processes in a chamber setting differ from those in the atmosphere, the calculation method must be adjusted for chamber experiments^84^. Specifically, the nucleation rate (*J*_1.7_) is calculated by factoring in losses specific to the CLOUD chamber, such as dilution, wall and coagulation losses. We calculated *J*_dp_ as follows:

$${J}_{1.7}=\frac{{\rm{d}}N}{{\rm{d}}t}+{S}_{{\rm{dil}}}+{S}_{{\rm{wall}}}+{S}_{{\rm{coag}}}$$

in which d*N*/d*t* is the time derivative of the total particle concentration above a certain particle size (here >1.7 nm for *J*_1.7_) and *S*_dil_, *S*_wall_ and *S*_coag_ are the particle losses owing to dilution, wall and coagulation. The details can be found in ref. ^84^. To calculate the coagulation sink, we used the combined particle-size distribution from three instruments (NAIS, nano-SMPS and long-SMPS).

Furthermore, the nucleation rate at 2.5 nm, *J*_2.5_, derived from the butanol CPC and corrected by the same method described above, is calculated. The results are presented in Extended Data Fig. 6 in the same format as in Fig. 2. Because the CPC was not affected by the systematic upgrade in the cooling system between CLOUD15 and CLOUD16, it serves to distinguish subtle changes in our data. For example, the nucleation rates from experiments with NO_*x*_ (filled squares in Fig. 2) seem to be similar to the experiments without NO_*x*_ (filled circles in Fig. 2). This is probably a result of systematic errors introduced by changing either the cooling system or the instrument-calibration experiments. On the other hand, Extended Data Fig. 6 shows that experiments with NO_*x*_ have nucleation rates higher than the experiments without NO_*x*_, therefore, isoprene nitrates (IP_1-2N_) do contribute, despite to a lesser extent compared with IP_0N_, to particle nucleation.

To calculate particle growth rates, we use the 50% appearance-time method, as outlined in previous studies^58,84,85^. It is worth noting that the appearance-time method can overestimate growth rates when the impacts of coagulation (coagulation sink, coagulation source and particle coagulation growth) are non-negligible compared with the condensation growth, but the coagulation impact is rather small in the CLOUD experiments. For a deeper understanding of the molecular-level theory behind the method, we refer to the theoretical derivation presented in ref. ^58^. The particle number size distribution data used to calculate growth rates between 3.2 and 8.0 nm are measured by the NAIS. During previous experiments with α-pinene and sulfuric acid, we have confirmed that the growth rates measured with the NAIS in total mode are similar to those measured with the DMA-train^86^.

### Comparison of experimental and ambient conditions

To compare the CLOUD experimental conditions with ambient conditions in the tropical upper troposphere, we summarize in Extended Data Table 1 the key chemical and physical parameters of the CLOUD experiments and the CAFE-Brazil (CB) flight campaign^13^. The CLOUD statistics are summarized from all experiments presented in this study, separated into two temperature conditions at −30 °C and −50 °C, respectively. Statistics of the CB flight campaign are derived from a single research flight, RF 19, samples T4 and T9, shown in Fig. 1 of ref. ^13^. All vapour concentrations are presented in units of molecules per cm^−^^3^—the quantities as measured—for both CLOUD and CB. The values from the CB campaign are not corrected to their values at standard temperature and pressure, to allow for a direct comparison of the chemical and aerosol formation kinetics between the CLOUD experiments and the flight measurements. We elaborate below on three aspects of this comparison: (1) isoprene non-nitrates (IP_0N_) and nitrates (IP_1-2N_); (2) atmospheric acids; and (3) impact of atmospheric pressure on particle nucleation.

#### Distribution of IP_0N_ and IP_1-2N_

In general, the CLOUD experiments were designed to mimic ambient conditions as closely as possible. Key parameters such as temperature, relative humidity (RH), isoprene, O_3_, NO and HO_2_/OH ratios are directly comparable between the CLOUD experiments and the CB measurements. The largest differences between CLOUD and CB are higher atmospheric pressure in CLOUD and higher OH/HO_2_ concentrations, resulting in a higher HO_2_/NO ratio in CLOUD. The higher OH concentration in the CLOUD chamber is required to reproduce ambient IP-OOM concentrations at a chamber-wall-loss rate of approximately 2 × 10^−3^ s^−1^. In the upper troposphere, the condensation sink for low-volatility gaseous species could be several times or even up to one order of magnitude lower than the chamber-wall-loss rate.

Because this study aims to investigate the contribution of IP_0N_ and IP_1-2N_ to particle nucleation and growth, these parameters are critical for CLOUD to reproduce at atmospheric concentrations. A consequence of the relatively higher HO_2_/NO ratio in CLOUD is that the IP_0N_ to IP_1-2N_ ratio is elevated compared with CB. However, because the higher operating pressure in CLOUD favours the formation of IP_1-2N_ by accelerating the reaction of organic peroxy radical (RO_2_) with NO as well as enhancing the organic nitrate formation branching ratio^35^ (see Extended Data Table 1), this effect largely compensates for the higher HO_2_/NO ratio in CLOUD. Nevertheless, regardless of the chemical details, which will be presented by follow-up studies, CLOUD successfully reproduces isoprene oxidation products IP_0N_ and IP_1-2N_, in terms of both absolute values and relative ratios (Extended Data Table 1). The wide range of IP_0N_ and IP_1-2N_ concentrations and IP_0N_/IP_1-2N_ ratios covered by CLOUD experiments enables CLOUD to reasonably simulate particle nucleation and growth dynamics, consistent with CB measurements during dawn hours (T4 period in Extended Data Table 1) and morning (T9 period in Extended Data Table 1). As the daylight hours proceed beyond the period measured by CB, both OH and HO_2_ concentrations will increase, whereas NO_*x*_ concentrations decrease, favouring the formation of IP_0N_ over IP_1-2N_. Thus, the importance of IP_0N_ may be further enhanced after noon. It is also noteworthy that chemical distribution and nucleation dynamics might differ in other seasons and locations from those covered by CB measurements, such as the cases presented in Fig. S11 of ref. ^5^. Therefore, we believe that the wide range of conditions explored by CLOUD provides valuable data to enable global models to evaluate the impact of isoprene on new particle formation in other upper-tropospheric environments in which NO_*x*_ concentrations may differ from those measured by CB.

#### Impact of sulfuric acid and iodine oxoacids

In this study, we observe a large enhancement of IP-OOM nucleation rates from trace amounts of atmospheric acids (specifically, H_2_SO_4_ and HIO_*x*_). The enhancement starts at acid concentrations of 10^5^ cm^−^^3^ and, at an acid concentration of 2 × 10^6^ cm^−^^3^, the particle-nucleation rate is approximately 100-fold faster than without added acids.

The CB measurements are unable to comment on this synergistic role of acids for IP-OOM particle nucleation owing to their H_2_SO_4_ detection limit of several times 10^6^ cm^−^^3^. Nevertheless, acid enhancement of IP-OOM nucleation can be expected to occur in the atmosphere. Aircraft measurements indicate that approximately 10 pptv SO_2_ is ubiquitous in the global atmosphere between the marine boundary layer and the upper troposphere^87,88^. Global simulations also suggest that H_2_SO_4_ concentrations greater than 10^5^ cm^−^^3^ are widespread throughout the troposphere^44^. The global distribution of HIO_*x*_ is less well known and global simulations and aircraft measurements are needed to quantify its concentrations in the upper troposphere. However, recent measurements of iodine oxide and particle-phase iodine in the upper troposphere^89^ suggest that HIO_*x*_ may also play a role in enhancing IP-OOM particle nucleation.

#### Impact of atmospheric pressure on particle nucleation

Cluster-forming interactions (such as nucleation) are of the form:

$$\begin{array}{ll}{\rm{A}}+{\rm{B}}\to {{\rm{C}}}^{* } & ({\rm{R}}1\,;\,{\rm{condensation}}),\\ {{\rm{C}}}^{* }\to {\rm{A}}+{\rm{B}} & ({\rm{R}}2\,;\,{\rm{evaporation}})\,{\rm{and}}\\ {{\rm{C}}}^{* }+{\rm{M}}\to {\rm{C}}+{\rm{M}} & ({\rm{R}}3\,;\,{\rm{thermalization}}),\end{array}$$

in which A and B are two molecules (or small clusters) forming the cluster C and C* is a vibrationally excited state of the cluster containing the cluster energy *E*_AB_ as part of its internal vibrational energy. The excited cluster C* will lose energy to the bath gas, M, with a concentration given by pressure through the ideal gas law. Cluster-forming interactions can therefore depend on ambient pressure^90^. When pressure is relatively ‘low’, reaction R3 will be the rate-limiting step for cluster formation and the overall rate will be of the third order (in A, B and M). However, when pressure is relatively high, reaction R1 will be the rate-limiting step and the rate will be of the second order (in A and B) and be independent of pressure, that is, at the so-called ‘high-pressure limit’. The critical pressure occurs when the rates of R2 and R3 are equal and therefore depends on the lifetime (evaporation rate) of C* through reaction R2, as well as the ambient pressure through reaction R3.

In practice, only systems with very few heavy atoms (four or fewer heavy atoms, in which ‘heavy’ excludes hydrogen) would show any meaningful pressure dependence in the atmosphere^91^, as quantified below. Because IP-OOM nucleation typically involves more than 15 heavy atoms, measurements in the CLOUD chamber at near 1 bar are directly applicable to the upper troposphere near 0.2 bar, provided the results are interpreted in terms of number concentrations (thus accounting for the dilution effect of reduced pressure) and not mixing ratios.

The (microcanonical) decomposition rates (inverse lifetimes) of the cluster are given by Rice–Ramsperger–Kassel–Marcus theory^90^. It depends strongly on the number of internal vibrational modes in C*. As a rule, it can be estimated (by Rice, Ramsperger and Kassel theory)^90^ roughly as the ‘fractional excess free energy’, $\upsilon {\left(\frac{e-{e}_{0}}{e}\right)}^{s}$, in which *υ* is a typical frequency, 100 THz or so, *e* is the cluster energy above the ground vibrational state and *e*_0_ is the critical energy for decomposition (the cluster energy *E*_AB_) and *s* is an effective number of vibrational modes in the cluster. Approximately, *e* − *e*_0_ will be on the order *kT* (200 cm^−^^1^), whereas for the systems nucleating under atmospheric conditions, *e*_0_ will be on the order 800 cm^−^^1^ or more. Thus, *e* will be on the order 1,000 cm^−^^1^ and (*e* − *e*_0_)/*e* will be on the order 0.2. Again, approximately and conservatively, *s* is 3*N* − 7, in which *N* is the number of heavy (non-H) atoms in C*. The ‘7’ excludes external modes as well as the reaction coordinate. A system with five heavy atoms would have a decay coefficient of roughly 2.6 × 10^8^ s^−^^1^, whereas the collision frequency at 1 atm is near 10^10^ s^−^^1^. Such a system would (barely) show some pressure dependence. By contrast, for isoprene oxidation products and H_2_SO_4_, *N* is probably 15, so *s* = 3*N* − 7 = 38. Given this, the microcanonical decay coefficients for these clusters will be on the order 3 × 10^−^^13^ s^−^^1^. This is extremely slow. In practice, it means that the energy distribution in cluster C will be entirely thermal, that is, given by a Boltzmann term at the ambient temperature, and the rates of cluster formation (and decomposition or evaporation) will be unaffected by pressure.

## Online content

Any methods, additional references, Nature Portfolio reporting summaries, source data, extended data, supplementary information, acknowledgements, peer review information; details of author contributions and competing interests; and statements of data and code availability are available at 10.1038/s41586-024-08196-0.

## Figures and Tables

**Extended Data Table 1:**
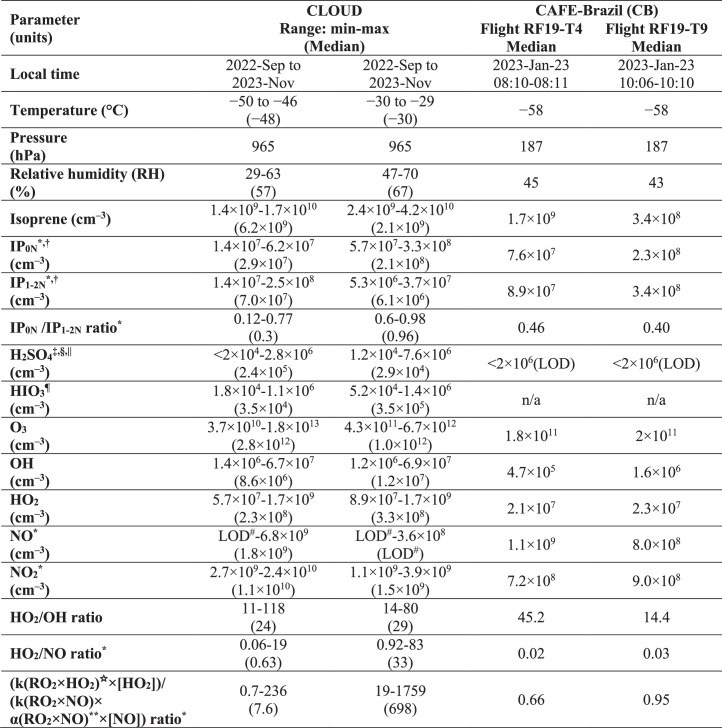
Comparison of CLOUD experimental conditions with the ambient conditions during CAFE-Brazil research flight RF 19, periods T4 and T9 (Curtius et al.^13^)

The times of the CB flights are in local time. *Only CLOUD experiments with added NO_*x*_. ^†^The definitions of IP_0N_ and IP_1-2N_ in the CB study refer to the measurement of the NO_3_^−^ chemical ionization alone, whereas the CLOUD results refer to measurements using the combination of NO_3_^−^, Br^−^ and NH_4_^+^ chemical-ionization methods. ^‡^Only CLOUD experiments with H_2_SO_4_. ^§^The CLOUD limit-of-detection (LOD) for H_2_SO_4_ is 2 × 10^4^ cm^−^^3^. ^||^The CB LOD for H_2_SO_4_ is 2 × 10^6^ cm^−^^3^ at altitudes above 8 km. ^¶^Only experiments with HIO_3_. ^#^The CLOUD LOD for NO is 2 × 10^8^ cm^−3^. ^☆^Using equation 7 in ref. ^35^. ^**^Using equations 1–4 in ref. ^35^.

## Data Availability

The full dataset shown in the figures and tables is available open access at 10.5281/zenodo.13736557 (ref.^92^).

## Code availability

Codes conducting the analysis presented here can be obtained on reasonable request to the corresponding authors.
